# Control of Type III Secretion System Effector/Chaperone Ratio Fosters Pathogen Adaptation to Host-Adherent Lifestyle

**DOI:** 10.1128/mBio.02074-19

**Published:** 2019-09-17

**Authors:** Netanel Elbaz, Yaakov Socol, Naama Katsowich, Ilan Rosenshine

**Affiliations:** a Department of Microbiology and Molecular Genetics, Institute of Medical Research Israel-Canada, Faculty of Medicine, The Hebrew University of Jerusalem, Jerusalem, Israel; University of Chicago

**Keywords:** enteropathogenic *Escherichia coli*, type III secretion system, Tir, CesT, CsrA

## Abstract

The transition from a planktonic lifestyle to a host-attached state is often critical for bacterial virulence. Upon attachment to host cells, enteropathogenic Escherichia coli (EPEC) employs a type III secretion system (T3SS) to inject into the host cells ∼20 effector proteins, including Tir. CesT, which is encoded from the same operon downstream of *tir*, is a Tir-bound chaperone that facilitates Tir translocation. Upon Tir translocation, the liberated CesT remains in the bacterial cytoplasm and antagonizes the posttranscriptional regulator CsrA, thus eliciting global regulation in the infecting pathogen. Importantly, tight control of the Tir/CesT ratio is vital, since an uncontrolled surge in free CesT levels may repress CsrA in an untimely manner, thus abrogating colonization. We investigated how fluctuations in Tir translation affect the regulation of this ratio. By creating mutations that cause the premature termination of Tir translation, we revealed that the untranslated *tir* mRNA becomes highly unstable, resulting in a rapid drop in *cesT* mRNA levels and, thus, CesT levels. This mechanism couples Tir and CesT levels to ensure a stable Tir/CesT ratio. Our results expose an additional level of regulation that enhances the efficacy of the initial interaction of EPEC with the host cell, providing a better understanding of the bacterial switch from the planktonic to the cell-adherent lifestyle.

## Abstract (precis)

Host colonization by extracellular pathogens often entails the transition from a planktonic lifestyle to a host-attached state. Enteropathogenic E. coli (EPEC), a Gram-negative pathogen, attaches to the intestinal epithelium of the host and employs a type III secretion system (T3SS) to inject effector proteins into the cytoplasm of infected cells. The most abundant effector protein injected is Tir, whose translocation is dependent on the Tir-bound chaperon CesT. Upon Tir injection, the liberated CesT binds to and inhibits the posttranscriptional regulator CsrA, resulting in reprogramming of gene expression in the host-attached bacteria. Thus, adaptation to the host-attached state involves dynamic remodeling of EPEC gene expression, which is mediated by the relative levels of Tir and CesT. Fluctuating from the optimal Tir/CesT ratio results in a decrease in EPEC virulence. Here we elucidate a posttranscriptional circuit that prevents sharp variations from this ratio, thus improving host colonization.

## INTRODUCTION

Most Escherichia coli strains are commensal nonpathogenic strains, yet some strains, including enteropathogenic E. coli (EPEC) and enterohemorrhagic E. coli (EHEC) strains, evolved into pathogens that cause conditions ranging from asymptomatic colonization to acute life-threatening disease (1–3). Both commensal and pathogenic E. coli strains colonize the intestinal epithelium; however, while commensal E. coli strains remain planktonic, residing within the mucous layer that lines the epithelium, the pathogens establish a cell-adherent lifestyle. The latter involves direct, tight, and lasting contact with the apical surface of the intestinal epithelial cells and the formation of attaching-and-effacing (A/E) lesions (4, 5). The transition of the pathogen from a planktonic to a cell-adherent lifestyle is dependent on a pathogenicity island termed the locus of enterocyte effacement (LEE), composed of a cluster of transcriptional units containing 41 genes encoding a type III secretion system (T3SS), six translocated effectors, and related proteins (4). Elucidating the processes that control this transition is critical for understanding the virulence of EPEC and EHEC.

Three proteins critical for establishing a cell-adherent lifestyle are clustered in the tricistronic operon *LEE5*, composed of *tir*, *cesT*, and *eae* (5). Tir is the most abundant effector (6, 7) and the first to be translocated into the host cell via the T3SS (8). The translocated Tir is inserted into the host cell membrane, assuming a hairpin topology with two transmembrane domains, a surface-exposed loop, and both termini residing in the host cell cytoplasm (9, 10). CesT is a T3SS chaperone that forms homodimers that bind two regions of Tir to form a complex with a 2:1 stoichiometry (11). The binding of Tir to CesT is critical for Tir stability in the bacteria and its translocation into the host cell via the T3SS (9, 11, 12). Although Tir is the major CesT binding partner, CesT binds and promotes the translocation of about 10 additional effectors, including Map, NleG, NleH1, NleH2, EspH, and EspZ (7, 8, 12, 13). Intimin, encoded by *eae*, is an outer membrane protein that binds specifically to the host surface-exposed loop of Tir, leading to tight bacterial attachment and clustering of Tir beneath the attached bacteria (14), triggering localized actin polymerization and formation of an actin structure termed pedestal (5, 15).

**FIG 1:**
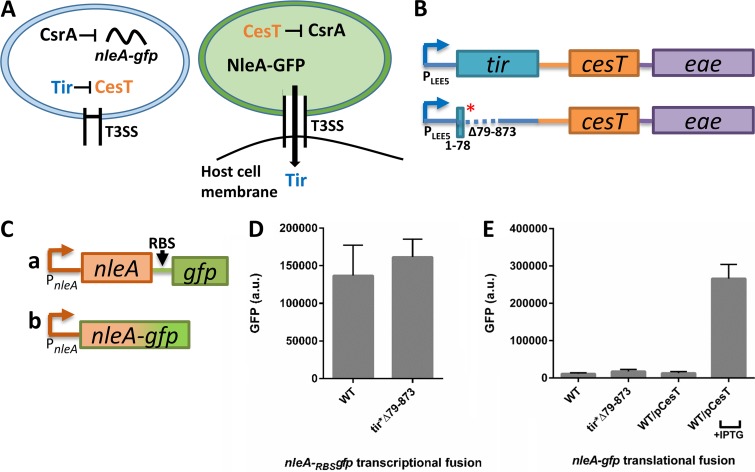
The EPEC tir*Δ79–873 mutant fails to express NleA-GFP. (A) The CesT-CsrA regulatory switch. In the preinfection state (left), CsrA binds to the *nleA* mRNA and represses its translation. In these cells, CesT is sequestered mainly by Tir. Upon attachment to the host and activation of the T3SS (right), Tir is translocated into the host cell and liberates CesT, which binds CsrA, thus promoting *nleA* translation. (B) Scheme of wild-type EPEC or tir*Δ79–873 mutant *LEE5* mRNA organization. Boxes represent translated genes, the red asterisk represents a stop codon, the dashed line represents a deletion, and solid lines represent untranslated regions. (C) Schematic representation of *nleA-gfp* transcriptional (row a) or translational (row b) fusions. RBS represents a synthetic ribosomal binding site. Both fusions are located at the native chromosomal location of *nleA*. The orange lines and arrows represent the native promoter and 5′ UTR of *nleA*. (D and E) Wild-type (WT) EPEC or the tir*Δ79–873 mutant containing the *nleA-gfp* transcriptional or translational fusion was statically grown overnight in LB and subcultured into DMEM for 5 h at 37°C to reach an OD_600_ of ∼0.6. The bacteria were then centrifuged and washed twice with PBS, and OD_600_-normalized GFP levels were determined by fluorimetry. As a positive control, we used wild-type EPEC supplemented with a plasmid expressing CesT under the regulation of the *lac* promoter (WT/pCesT). In this case, to stimulate CesT expression, IPTG (0.5 mM) was added. Error bars represent the standard deviation from three independent experiments. a.u., arbitrary units.

We and others recently found that following Tir translocation into the host, CesT is liberated in the EPEC cytoplasm, enabling its binding to CsrA, thus antagonizing its function (Fig. 1A) (6, 16). CsrA is a conserved RNA-binding protein that posttranscriptionally regulates hundreds of E. coli genes and that is consequently the regulator of multiple physiological processes, including carbon metabolism, production of secondary metabolites, motility, biofilm formation, and virulence (17, 18). The CesT-CsrA interaction leads to a major reprogramming of gene expression in the attached pathogen (6). Notably, among the genes that are positively regulated by CsrA are genes encoding the type 4 bundle-forming pilus (BFP), which promotes microcolony formation and host attachment (6, 19–21), and *LEE4*, encoding *espA*, *espB*, and *espD* (22), which are essential for injection of effector proteins into the host cell. CsrA binds to the *LEE4* mRNA to activate its translation (22, 23). As a result, repression of CsrA activity by CesT leads to reduced host attachment and T3SS activity (6). These findings highlight the notion that the optimal levels of CesT in the bacterium must be tightly and dynamically controlled to allow proper T3SS activity and the transition to the host-attached state. Untimely increased levels of effector-free CesT in planktonic EPEC might lead to the suppression of T3SS activity by antagonizing CsrA, yet too little CesT could lead to reduced effector translocation. Hence, significant fluctuations from optimal CesT levels might lead to reduced host colonization. However, it is only partially understood how optimal levels of CesT are maintained in the infecting EPEC.

In this study, we explored how EPEC adjusts its CesT levels in the face of perturbations in the levels of Tir, its major binding partner. Perturbations in Tir levels might arise from translational errors due to ribosome stalling or ribosome sliding on stretches of homopolymeric mRNA sequences (24), which are abundant in the AT-rich *tir* gene. A different type of translation perturbation is mediated by small RNAs (sRNAs) (25, 26), and if this is the case, a yet to be defined sRNA might specifically repress Tir translation, thus influencing the Tir/CesT ratio. Our analysis exposed a coordinated posttranscriptional control of the Tir/CesT ratio, ensuring the dynamic adjustment of CesT levels to optimize T3SS activity. Thus, this study elucidates a new layer of the intricate multilevel circuit that controls the transition of major pathogens from the planktonic to the host-attached state.

## RESULTS

### The EPEC tir*Δ79–873 mutant fails to activate NleA expression.

Since Tir is the most abundant CesT-binding effector (6, 7), perturbations in Tir expression might mimic Tir elimination by secretion, leading to increased levels of free CesT and, thus, to premature CsrA repression (Fig. 1A). To test this possibility, we employed an EPEC mutant in which *tir* was inactivated, thus simulating abrogation of Tir expression. This mutant, described by Kenny et al. (10), contains a stop codon after base pair (bp) 78 of the *tir* open reading frame (ORF) and a large internal deletion from positions 79 to 873 (Fig. 1B). This mutant is here designated tir*Δ79–873. As a readout for repression of CsrA by CesT, we monitored the production of NleA, whose translation is repressed by CsrA (Fig. 1A) (6). To this end, we used wild-type EPEC and the tir*Δ79–873 mutant, supplemented with chromosomal *nleA-gfp*, by forming either transcriptional fusions (*nleA-*_RBS_*gfp*, where _RBS_*gfp* represents the gene for green fluorescent protein [GFP] with a synthetic ribosomal binding site [RBS]) or translational fusions (*nleA-gfp*) (Fig. 1C). Strains were grown to a mid-logarithmic phase in Dulbecco’s modified Eagle medium (DMEM), and the levels of GFP synthesis in the cultures were determined by fluorimetry. Unexpectedly, the tir*Δ79–873 mutant showed a wild-type phenotype, expressing *nleA* mRNA yet failing to produce NleA-GFP (Fig. 1D and E). As a positive control, we examined wild-type EPEC overexpressing CesT, previously shown to trigger NleA translation (Fig. 1E) (6). These findings indicate that abrogating Tir translation does not result in the CesT-dependent repression of CsrA.

### EPEC tir*Δ79–873 contains low levels of CesT.

**FIG 2:**
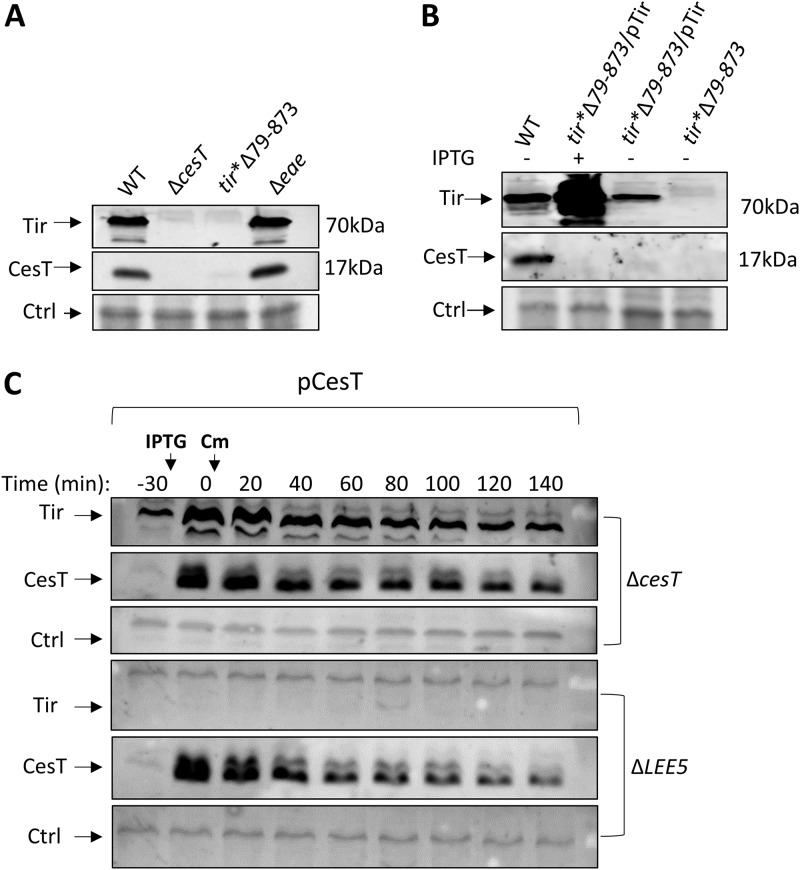
Link between Tir and CesT expression. (A) The EPEC tir*Δ79–873 mutant produces low levels of CesT. Wild-type (WT) EPEC and mutants, including the Δ*cesT*, tir*Δ79–873, and Δ*eae* mutants, were statically grown overnight in LB, subcultured in DMEM, and grown for 5 h to an OD_600_ of ∼0.6. The proteins were then extracted, and normalized bacterial extracts were analyzed by Western blotting using an anti-CesT/anti-Tir antibody. Nonspecific bands were used as a loading control (Ctrl). (B) *trans* complementation of tir*Δ79–873 mutant by *tir.* Wild-type EPEC or the tir*Δ79–873 and tir*Δ79–873/pTir mutants were statically grown overnight in LB and subcultured in DMEM for 3 h. IPTG (0.5 mM) was then added, and the bacteria were allowed to grow to an OD_600_ of ∼0.6. The bacterial fractions were harvested, and normalized bacterial extracts were analyzed by Western blotting using an anti-CesT/anti-Tir antibody. Nonspecific bands were used as a loading control. (C) Tir is not required for CesT stability. EPEC Δ*LEE5* or Δ*cesT* was supplemented with a plasmid expressing CesT via the *lac* promoter (pCesT). The strains were statically grown overnight in LB and subcultured into DMEM for 3 h, followed by IPTG addition (0.5 mM) (time, −30 min) to induce CesT expression. After 30 min, chloramphenicol (100 μg/ml) was added to block protein synthesis, and the levels of CesT and Tir were tracked over time by Western blotting using anti-CesT/anti-Tir antibody. Time points are indicated above the blot. Nonspecific bands were used as a loading control.

Since *cesT* is located downstream of *tir*, we compared the levels of CesT and Tir in wild-type EPEC to those in the tir*Δ79–873 mutant, using a polyclonal antibody that recognizes both Tir and CesT (see Fig. S1 in the supplemental material). The results showed that Tir was abolished in the Δ*cesT* mutant (Fig. 2A), likely due to its previously reported reduced Tir stability in the absence of CesT (9). Importantly, the tir*Δ79–873 mutant exhibited a marked reduction of CesT levels, whereas deletion of *eae* had no such effect (Fig. 2A). Presumably, the reduced levels of CesT diminished the CesT-mediated repression of CsrA, explaining the failure of the tir*Δ79–873 mutation to cause activation of NleA translation (Fig. 1E).

10.1128/mBio.02074-19.1 FIG S1 Rabbit polyclonal anti-CesT/Tir antibody. E. coli DH5α transformed with pTir or pCesT was grown with agitation in LB at 37°C to an OD_600_ of ∼0.6 in the presence or absence of 0.5 mM IPTG to induce Tir or CesT expression. The bacteria were harvested, and normalized bacterial extracts were analyzed by Western blotting using anti-CesT/anti-Tir antibody. Download FIG S1, TIF file, 0.4 MB. Copyright © 2019 Elbaz et al. 2019 Elbaz et al. This content is distributed under the terms of the Creative Commons Attribution 4.0 International license.

### Tir is not required for CesT stability.

The reduction in CesT levels in the tir*Δ79–873 mutant may indicate that the interaction between Tir and CesT promotes CesT stability. To address this possibility, we complemented the tir*Δ79–873 mutant with a plasmid expressing Tir and examined whether it restored the levels of CesT. The results showed that CesT levels remained low even upon supplementation of Tir in *trans* (Fig. 2B), suggesting that Tir is not required for CesT stability. To further support this conclusion, a direct comparison of CesT stability in the presence or absence of Tir was carried out. To this aim, an EPEC Δ*cesT* or Δ*LEE5* mutant supplemented with a CesT-expressing plasmid was treated with a translation inhibitor and the CesT degradation rate was tracked over time. Confirming the results presented above (Fig. 2B), no significant difference in the degradation rate of CesT was observed in the presence or the absence of Tir (Fig. 2C). Taken together, these results indicate that the tir*Δ79–873 mutation influences the levels of CesT in *cis*, regardless of the presence of the Tir protein.

### Translation of *tir* is required for CesT production.

**FIG 3:**
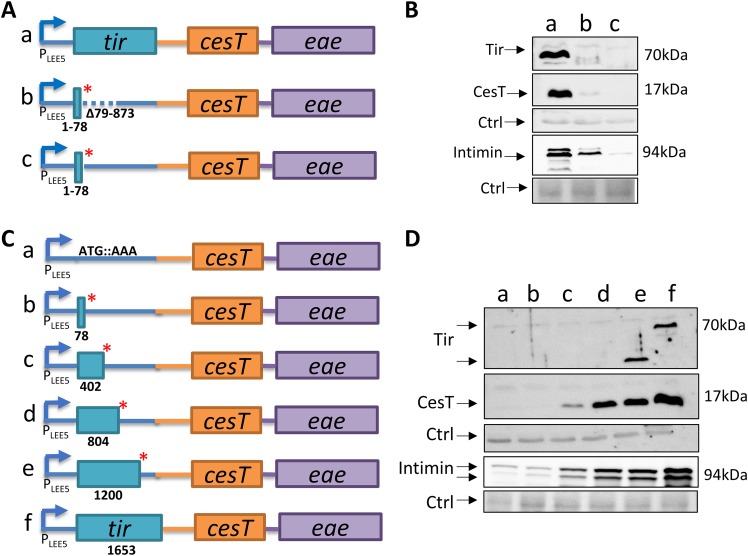
*tir* translation is required for expression of *LEE5* genes. (A) Scheme depicting the chromosomal *LEE5* organization of wild-type EPEC (row a) and the tir*Δ79–873 (row b) and tir*78 (row c) mutants. Boxes represent translated genes, the red asterisk represents a stop codon, the dashed line represents a deletion, and solid lines represent untranslated regions. (B) The EPEC strains in lanes a, b, and c, corresponding to rows a, b, and c in panel A, respectively, were grown in DMEM to an OD_600_ of ∼0.6 (∼5 h) and harvested. Normalized bacterial extracts were then analyzed by Western blotting using anti-CesT/anti-Tir and anti-intimin antibodies. Nonspecific bands were used as a loading control (Ctrl). (C) Schematic representation of chromosomal *LEE5* in strains containing inserted stop codons along the *tir* sequence, including the tir*1 (*tir*ATG::AAA), tir*78, tir*402, tir*804, tir*1200, and wild-type EPEC strains (rows a, b, c, d, e, and f, respectively). The numbers represent the nucleotide number followed by a stop codon. (D) The strains in lanes a to f, corresponding to those shown in rows a to f of panel C, respectively, were grown in DMEM to an OD_600_ of ∼0.6, and normalized bacterial extracts were analyzed by Western blotting using anti-CesT/anti-Tir and anti-intimin antibodies. Nonspecific bands were used as a loading control. The corresponding bands are indicated by arrows. For Tir, the upper arrow points to full-size wild-type Tir, seen only in lane f. The lower arrow points to a truncated Tir, seen only in lane e. The shorter forms of Tir in all other lanes were not detected by the antibody.

To better describe how the tir*Δ79–873 mutation affects CesT levels, we inserted a stop codon into the native chromosomal *tir* gene after bp 78 (tir*78) without deleting any *tir* coding sequence (Fig. 3A). Notably, in this mutant, the CesT levels were even lower than the levels observed in the tir*Δ79–873 mutant (Fig. 3B), suggesting that Tir translation is required for CesT production. For a deeper examination of the relationship between Tir translation and CesT production, we generated a set of chromosomal EPEC mutants by eliminating the first codon (*tir*ATG::AAA) or inserting a stop codon at positions 78, 402, 804, and 1200 along the *tir* coding region (Fig. 3C). We then examined the production of Tir, CesT, and intimin by these mutants. The results showed a major reduction in CesT levels upon the complete annulment of *tir* translation, as well as a gradual restoration of CesT production in correlation with an increase in the size of the translated Tir fragment (Fig. 3D). Production of intimin showed a pattern similar to that observed for CesT (Fig. 3D). Notably, a similar coupling between *tir* translation and CesT production was observed in the *LEE5* operons of EHEC and Citrobacter rodentium (Fig. S2), indicating that this coupling is a conserved feature in attaching-and-effacing pathogens. Taken together, these results suggest that *tir* translation may be required for the stability of the *LEE5* mRNA.

10.1128/mBio.02074-19.2 FIG S2 Translation of *tir* is required for CesT production by A/E pathogens. (A) Scheme of plasmids containing *LEE5* derivatives that originated from the genomes of Citrobacter rodentium or enterohemorrhagic E. coli (EHEC). In both cases, the *tir-cesT* region (blue-orange fragment) was cloned between the synthetic promoter-5′ UTR (red fragment and arrow) and *gfp* (green), which, together with *tir* and *cesT*, form a tricistronic operon. Boxes represent translated genes, and solid lines represent untranslated regions. For each pathogen, *tir* remained native or the first ATG codon was replaced by AAA (ATG::AAA). (B) E. coli K-12 (MG1655) was transformed with the indicated plasmids. The bacteria were grown in LB at 37°C to an OD_600_ of ∼0.6. Then, the bacterial cultures were harvested and normalized bacterial extracts were analyzed by Western blotting using anti-CesT antibody. Nonspecific bands were used as a loading control (Ctrl). (C) C. rodentium (CR) or EHEC was transformed with the mentioned plasmids. The bacteria were then statically grown overnight in LB and subcultured in DMEM to an OD_600_ of ∼0.6. The bacteria were then harvested, and GFP levels were determined by fluorimetry. Error bars represent the standard deviation from three biological repeats done in triplicate. Download FIG S2, TIF file, 0.8 MB. Copyright © 2019 Elbaz et al. 2019 Elbaz et al. This content is distributed under the terms of the Creative Commons Attribution 4.0 International license.

### Translation of *tir* is required for the stability of *LEE5* mRNA.

**FIG 4:**
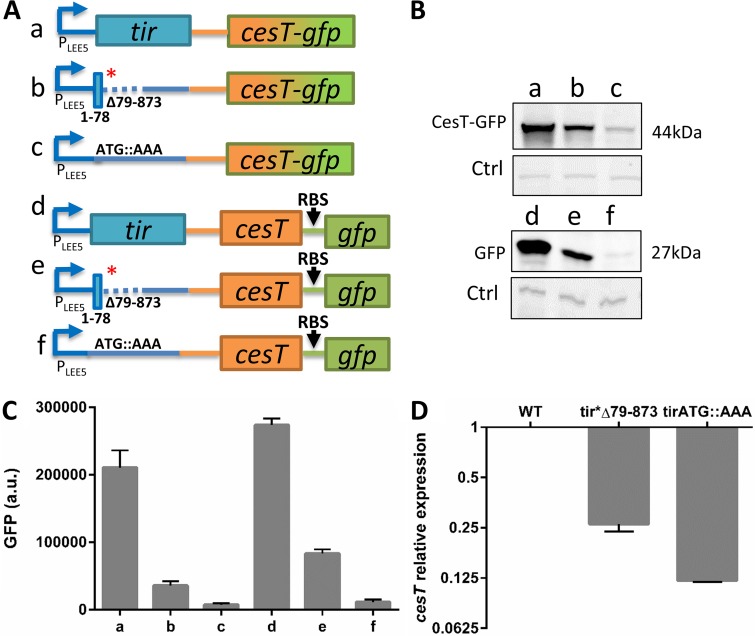
Obstruction of *tir* translation results in reduced levels of *LEE5* mRNA. (A) Schematic illustration of the chromosomal *LEE5* region of EPEC strains harboring wild-type *tir* or the tir*Δ79–873 or *tir* ATG::AAA mutant. These strains also contain the *cesT-gfp* translational fusion (rows a, b, and c, respectively) or transcriptional fusion (rows d, e, and f, respectively). (B, C) The strains in lanes a to f, corresponding to rows a to f of panel A, respectively, were grown in DMEM to an OD_600_ of ∼0.6 and harvested. Normalized bacterial extracts were analyzed by Western blotting using anti-GFP antibody (B). Nonspecific bands were used as a loading control (Ctrl). Alternatively, GFP levels in intact cultures were determined by fluorimetry (C). Error bars represent the standard deviation from three independent experiments done in triplicate. (D) RNA was extracted from wild-type (WT) EPEC and the tir*Δ79–873 and *tir* ATG::AAA mutants, and the amount of *cesT* transcript was analyzed by qPCR using the primers indicated in Table S2 in the supplemental material. Error bars represent standard deviations.

To examine whether *tir* translation affects the amount of *LEE5* mRNA, we created a set of chromosomal fusions of *cesT* and *gfp*, including translational (*cesT-gfp*) and transcriptional (*cesT*-_RBS_*gfp*) fusions. As parental strains, we used wild-type EPEC and EPEC containing the tir*Δ79–873 or *tir*ATG::AAA mutation (Fig. 4A). We then examined these strains for the expression of GFP or CesT-GFP by Western blotting analysis using an anti-GFP antibody and fluorimetry. The results showed a similar reduction in GFP and CesT-GFP when *tir* was not translated (Fig. 4B and C), supporting the notion that when *tir* mRNA is not translated, the levels of *LEE5*-*cesT* mRNA are reduced. To directly test this prediction, we examined the levels of *tir* and *cesT* mRNA in the three parental EPEC strains using quantitative PCR (qPCR). In agreement with the data presented above, we recorded a marked reduction in *cesT* and *tir* mRNA levels when *tir* was not translated (Fig. 4D; Fig. S3). These results suggest that, when not translated, the exposed *tir* mRNA promotes the rapid degradation of the *LEE5* transcript.

10.1128/mBio.02074-19.3 FIG S3 Obstruction of *tir* translation results in reduced levels of *LEE5* mRNA. Total RNA was extracted from wild-type (WT) EPEC and EPEC mutants in which the chromosomal *tir* contained either the elimination of the first codon (ATG::AAA) or a stop codon at position 804 (804*). The amount of *tir* mRNA was evaluated by qPCR using the primers indicated in Table S2 in the supplemental material. Error bars represent the standard deviation from three biological repeats done in triplicate. Download FIG S3, TIF file, 0.5 MB. Copyright © 2019 Elbaz et al. 2019 Elbaz et al. This content is distributed under the terms of the Creative Commons Attribution 4.0 International license.

10.1128/mBio.02074-19.8 TABLE S2 Primers and antibodies used in this study. Download Table S2, DOCX file, 0.02 MB. Copyright © 2019 Elbaz et al. 2019 Elbaz et al. This content is distributed under the terms of the Creative Commons Attribution 4.0 International license.

### Degradation of untranslated *tir* mRNA occurs in E. coli K-12.

**FIG 5:**
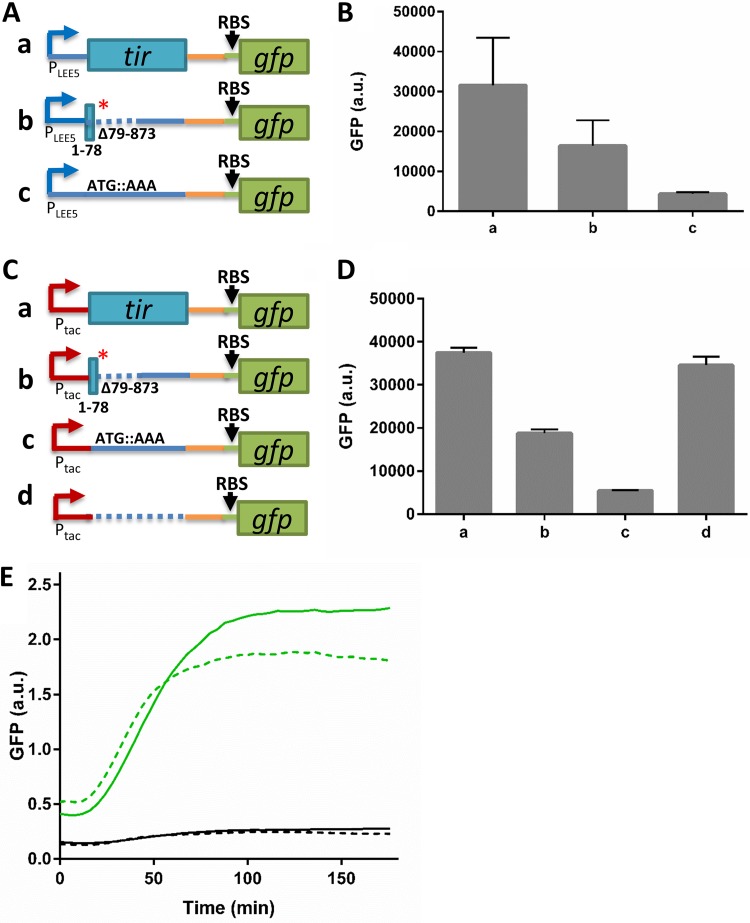
Deletion of the *tir* ORF restored *LEE5* expression levels. (A) Scheme of plasmids containing derivatives of *tir* and flanking regions, including the entire *tir-cesT* intergenic region (solid orange line) transcriptionally fused to *gfp*. These plasmids are expressed via the native promoter and 5′ UTR (blue arrows). Boxes represent translated genes, the red asterisk represents a stop codon, the dashed line represents a deletion, and solid lines represent untranslated regions. (B) E. coli K-12 (MG1655) was transformed with the plasmids shown in panel A (with columns a to c corresponding to rows a to c in panel A, respectively). The strains were further transformed with a compatible plasmid encoding Ler, the positive regulator of the *LEE5* promoter. Bacteria were grown to an OD_600_ of ∼0.6 in M9 medium, supplemented when needed with 0.05 mM IPTG to induce Ler expression. OD_600_-normalized GFP levels were determined by fluorimetry. Error bars represent the standard deviation from three independent experiments done in triplicate. (C) Scheme of plasmids containing derivatives of *tir* and flanking regions (rows a to c) or lacking *tir* (row d), including the entire *tir-cesT* intergenic region (solid orange lines) transcriptionally fused to *gfp*. All plasmids are expressed via the *tac* promoter and *tac* 5′ UTR (red arrow). Ribosomal binding sites (RBS) are indicated, boxes represent translated genes, the red asterisk represents a stop codon, the dashed lines represent a deletion, and solid lines represent untranslated regions. (D) E. coli K-12 (MG1655) was transformed with the plasmids shown in panel C (with columns a to d corresponding to rows a to d in panel C, respectively). The bacteria were then grown in M9 medium, and OD_600_-normalized GFP levels were determined by fluorimetry. Error bars represent the standard deviation from three independent experiments done in triplicate. (E) E. coli K-12 (MG1655) or an isogenic strain containing the *rne3071* mutation (an RNase E temperature-sensitive mutant [RNase E^ts^]) were transformed with the plasmids shown in panel C, rows a and c. The bacteria were then grown overnight in LB (30°C), subcultured in M9 medium, and grown at 30°C to an OD_600_ of 0.3, and then the temperature was shifted to 42°C for 30 min. Next, IPTG (0.25 mM) was added, and normalized GFP expression levels, reflecting the amount of *gfp* mRNA, were monitored by fluorimetry in real time at 5-min intervals. Bacteria containing the plasmids in rows a and c of panel C are shown in green and black lines, respectively. Solid lines represent the RNase E^ts^ mutant, and dashed lines represent wild-type bacteria. Shown is the average of quadruplicates. The SD was typically <0.12; bars are not shown for clarity.

We next tested whether an EPEC-specific factor is responsible for the *LEE5* mRNA instability in the absence of *tir* translation. To this aim, we cloned the wild-type *tir*, the tir*Δ79–873, and the *tir*ATG::AAA alleles and their flanking sequences, including the regulatory regions and 3′ untranslated region (UTR) fused to _RBS_*gfp* (Fig. 5A). We introduced these plasmids into E. coli K-12 (MG1655), and to facilitate the activation of the *LEE5* promoter, the strains were supplemented with a compatible plasmid expressing Ler, a positive regulator of the *LEE5* promoter (27). We then examined the GFP levels generated by these strains. Consistent with the results shown in Fig. 4, when *tir* was not translated, we noted a dramatic reduction in GFP synthesis, as evidenced by reduced fluorescence (Fig. 5B), reflecting lower levels of mRNA. These results indicate that specific EPEC factors are not required for the degradation of the *LEE5* mRNA, further confirming that *tir* translation is required for maintaining high levels of *LEE5* mRNA, and indicate that the cloned fragment contains all the sequences required for this regulation.

### *tir* mRNA contains sequences that promote instability in the absence of translation.

We hypothesized that the coding region of *tir* mRNA is specifically targeted by RNases but that translation protects it from degradation. This prediction implies that deletion of the entire *tir* ORF should restore the mRNA stability of *LEE5* including that of *cesT*. To examine this prediction, we first aimed to exclude the possibility that the tempered activity of the *LEE5* promoter is involved in the reduced *cesT* mRNA levels shown in Fig. 4D and 5B. We thus replaced the native *LEE5* promoter and the *tir* 5′ UTR of the plasmids shown in Fig. 5A with a synthetic *tac* promoter and 5′ UTR (Fig. 5C, rows a to c) and then tested for GFP production. As expected, the results showed a similar profile regardless of the promoter and 5′ UTR identity (Fig. 5D, columns a to c). We next examined a derivative of these plasmids containing the *tac* promoter and *tac* 5′ UTR but lacking the entire *tir* coding region (Fig. 5C, row d) and tested the influence of this deletion on the stability of the mRNA using GFP synthesis as a readout for mRNA levels. The results showed that deletion of the entire *tir* coding region restored GFP production levels to those obtained with the wild-type *tir*-containing plasmid (Fig. 5D, column d), supporting the premise that the untranslated *tir* mRNA is unstable.

### RNase E is not required for *LEE5* mRNA instability in the absence of *tir* translation.

Since bacterial RNA degradation is mediated mainly by RNase E (28), we examined whether RNase E mediates the degradation of an untranslated *LEE5* transcript. To this end, the plasmids whose constructs are shown in Fig. 5C, rows a and c, were transformed into wild-type E. coli K-12 (MG1655) and into an isogenic RNase E temperature-sensitive (RNase E^ts^) mutant (the *rne3071* mutant) (29). The bacteria were then grown under permissive conditions (30°C) to an optical density at 600 nm (OD_600_) of 0.3, followed by a temperature shift (42°C) to inactivate the RNase E. Next, IPTG (isopropyl-β-d-thiogalactopyranoside) was added to induce the transcription of the *tir*-*gfp* bicistronic operon. The GFP production levels, which reflect the levels of the transcript, were tracked over time using fluorimetry. The results showed that the level of untranslated *tir-gfp* remained very low even upon inactivation of RNase E (Fig. 5E). Thus, the identity of the RNase responsible for the degradation of the untranslated *LEE5* transcript remains to be defined.

### The *tir* 5′ UTR and 5′ coding region are required for efficient *LEE5* transcription.

**FIG 6:**
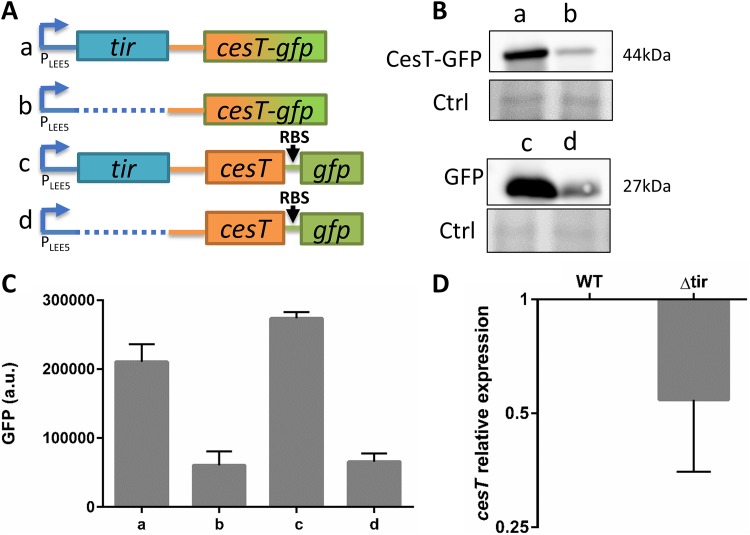
A *tir* coding region is required for efficient *LEE5* transcription. (A) Schematic presentation of the constructed chromosomal *tir-cesT* region containing intact *tir* (rows a and c) or lacking *tir* (Δ*tir*; rows b and d), as well as a translational or a transcriptional *cesT-gfp* fusion, as indicated. Ribosomal binding sites (RBS) are indicated, and boxes represent translated genes, and dashed lines represent a deletion. (B, C) The strains in lanes a to d, corresponding to those shown in rows a to d of panel A, respectively, were grown in DMEM to an OD_600_ of ∼0.6 and harvested. Normalized bacterial extracts were then analyzed by Western blotting using anti-GFP antibody (B). Nonspecific bands were used as a loading control (Ctrl). Alternatively, the levels of GFP in intact cultures were determined by a fluorimetry (C). (D) RNA was extracted from wild-type EPEC and the Δ*tir* mutant, and the amount of *cesT* transcript was analyzed by qPCR using the primers indicated in Table S2 in the supplemental material. Error bars represent the standard deviation.

Given the results shown in Fig. 5D, we hypothesized that deletion of the chromosomal *tir* ORF (Δ*tir*) would restore the stability of the *LEE5* or *cesT* mRNA. To test this prediction, we constructed suitable strains containing modified chromosomal *LEE5* operons (Fig. 6A). We then compared the levels of CesT and *cesT* mRNA in the wild-type EPEC strain and the Δ*tir* mutant. Surprisingly, in contrast to our prediction, we found reduced levels of both CesT-GFP and *cesT* mRNA in EPEC Δ*tir* (Fig. 6B to D). These results suggest the existence of an additional regulatory layer controlling *LEE5* expression.

To investigate whether this secondary regulation acts at the transcriptional or translational level, we utilized a set of plasmids with or without the *tir* ORF linked to its native promoter-5′ UTR or to a synthetic promoter-5′ UTR (Fig. S4A, rows a to d). These plasmids were introduced into E. coli K-12 (MG1655), which, if needed, was supplemented with a compatible plasmid expressing Ler. We then examined the GFP levels generated by these plasmids, and in agreement with the results shown in Fig. 6B to D, we noted a significant reduction in the levels of GFP when the native promoter and 5′ UTR were used but the *tir* coding region was removed (Fig. S4A, rows a and b), indicating lower levels of *LEE5* mRNA in the absence of the *tir* coding region (Fig. S4B and C, columns a and b). In contrast, GFP levels remained unaffected, regardless of the presence of the *tir* ORF, when the *tac* promoter and synthetic 5′ UTR were used (Fig. S4B and C, columns c and d). These results indicate that the activity of the native *LEE5* promoter is enhanced by a sequence within the *tir* coding region.

10.1128/mBio.02074-19.4 FIG S4 The *tir* native 5′ UTR and coding region are required for transcription via the native *LEE5* promoter. (A) Scheme of plasmid-borne *tir* and flanking regions, all of which contained the 3′ UTR transcriptionally fused to *gfp*. In rows b, d, f, and h, the *tir* ORF was deleted, and in rows c and d, the native promoter and 5′ UTR (blue arrow and line, respectively) were replaced by a synthetic one (P*tac*) (red arrow and line). The plasmids in rows e to h are chimeric promoter-5′ UTR plasmids. The plasmids in rows e and f contain the *LEE5* promoter and *tac* 5′ UTR, respectively. The plasmids in rows g and h contain the *tac* promoter and the *tir* 5′ UTR, respectively. (B) E. coli K-12 (MG1655) was transformed with the plasmids in rows a to h of panel A, and strains containing the plasmids in rows a, b, e, and f were further supplemented with a plasmid encoding Ler to induce *LEE5* expression. The bacteria were grown in LB to an OD_600_ of ∼0.6 and supplemented when needed with 0.05 mM IPTG to induce Ler expression, Cells were harvested, and normalized extracts were analyzed by Western blotting using anti-Tir and anti-GFP antibodies. Nonspecific bands were used as loading controls (Ctrl). (C) The strains whose constructs are shown in panel A were grown overnight in LB and subcultured in M9 medium, which was supplemented, when needed, with 0.05 mM IPTG to induce Ler expression, and normalized GFP levels were determined by fluorimetry. Error bars represent the standard deviation from three independent experiments done in triplicate. Download FIG S4, TIF file, 1.6 MB. Copyright © 2019 Elbaz et al. 2019 Elbaz et al. This content is distributed under the terms of the Creative Commons Attribution 4.0 International license.

To investigate whether the reduced *LEE5* transcription caused by the absence of the *tir* coding region is dependent on the *LEE5* promoter or its 5′ UTR, we created a set of plasmids containing a chimeric promoter-5′ UTR (Fig. S4A, rows e to h). The results showed that the *tac* promoter stimulates similar levels of expression of *LEE5* regardless of the presence of the *tir* ORF or the 5′ UTR identity (Fig. 5C and D and Fig. S4B and C, columns e to h). In contrast, the native *LEE5* promoter became functional only when linked to its native 5′ UTR, followed by the *tir* coding region (Fig. S4B and C). Taken together, the results presented above suggest that transcription via the native *LEE5* promoter requires DNA sequences overlapping its native 5′ UTR and the 5′ region of the *tir* coding sequence.

### Low CesT levels upon a reduction in Tir translation lead to infection deficiency.

**FIG 7:**
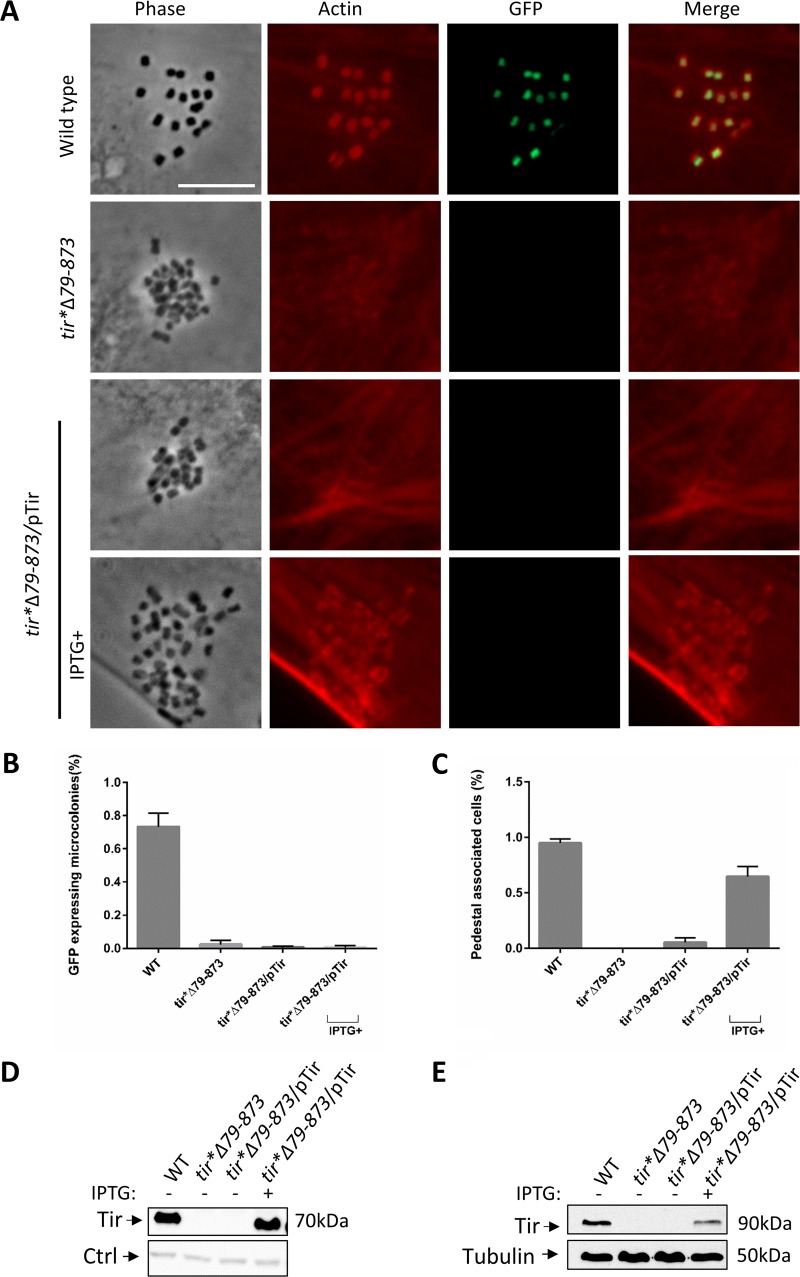
Complementation of the tir*Δ79–873 mutant fails to restore NleA-GFP production and pedestal formation by attached bacteria. (A) HeLa cells were infected for 3 h with wild-type EPEC or mutants, including the tir*Δ79–873 mutant, the tir*Δ79–873/pTir mutant, or the tir*Δ79–873/pTir mutant supplemented with 0.5 mM IPTG to induce Tir production. All strains contained the genomic *nleA-gfp* translational fusion. The infected cells were fixed and stained with phalloidin rhodamine (which stains actin; red), and images were recorded. Bar, 10 μm. (B) HeLa cells were infected as described in the legend to panel A, and the percentage of attached microcolonies (*n* > 100) that expressed NleA-GFP was determined. Error bars represent the standard deviation from three independent experiments. (C) HeLa cells were infected as described in the legend to panel A, and the percentage of cells (*n* > 100) that contained pedestals (*n* > 20) was determined. Error bars represent the standard deviation from three independent experiments. (D) The indicated strains were statically grown in DMEM for 3 h at 37°C to an OD_600_ of ∼0.2, with or without the addition of IPTG (0.5 mM), and bacteria were allowed to grow to an OD_600_ of ∼0.6. Bacterial fractions were analyzed by Western blotting using anti-Tir antibody. A nonspecific band recognized by the antibody was used as a loading control (Ctrl). (E) HEK293T cells were infected with the indicated strains for 3 h, washed, scraped, lysed, and subjected to centrifugation. The resulting supernatants containing translocated Tir were analyzed by Western blotting using anti-Tir antibody. Tubulin served as a loading control.

Our results suggest that a drop in CesT levels upon a reduction in *tir* translation would lead to reduced translocation of the CesT-dependent effectors even upon supplementation of Tir in *trans*. To examine this notion, we compared HeLa cells infected with wild-type EPEC or with the tir*Δ79–873 mutant. Both bacterial strains were supplemented with the chromosomal *nleA-gfp* translational fusion (Fig. 1C, row b), allowing the use of microscopy for assessment of CesT-mediated repression of CsrA in individual host-attached bacteria. In addition, a plasmid expressing wild-type Tir was introduced into the tir*Δ79–873 mutant, and the Tir-dependent formation of actin-pedestal was used as a readout for CesT-dependent Tir translocation. The infected cells were fixed, stained for actin, and examined by microscopy. The results showed that wild-type EPEC exhibited an efficient pedestal formation associated with the marked activation of NleA-GFP expression (Fig. 7A to C). The pedestal formation indicates efficient Tir translocation, and activation of NleA-GFP expression reports efficient repression of CsrA by the liberated CesT. In contrast, the tir*Δ79–873 mutant failed to show activation of *nleA-gfp* expression (Fig. 7A and B) and exhibited a reduced translocation of Tir when supplemented in *trans* (Fig. 7D and E). Furthermore, the formation of actin pedestals was only partially restored in the Tir-complemented mutant, as fewer pedestals were formed, and the ones that were formed appeared faint, diffuse, and truncated (Fig. 7A and C). Similar experiments were repeated with mutants containing increasing lengths of *tir* translation (Fig. 3C) and showed a gradual reestablishment of *tir* stability (Fig. S5A) and actin pedestal formation (Fig. S5B and C), which correlates with the size of the *tir* translated sequence and the production levels of CesT (Fig. 3D and Fig. S5). In the *tir*ATG::AAA mutant, we also noted a reduction in the level of production and secretion of the translocon proteins EspA and EspB as well as the Map effector (Fig. S6), possibly due to changes in the CesT/CsrA ratio. Taken together, our results show that perturbations in *tir* translation cause the rapid degradation of *LEE5* mRNA, including that of *cesT*, leading to a drop in CesT levels and a reduction in virulence. The reduced infectivity is likely due to reduced CesT-dependent effector translocation and the deregulated activity of CsrA.

10.1128/mBio.02074-19.5 FIG S5 Pedestal formation by a set of *tir* mutants containing stop codons. Wild-type EPEC or mutants containing stop codons along the *tir* sequence (the *tir*ATG::AAA, tir*78, tir*402, tir*804, and tir*1200 mutants) were transformed with a Tir-expressing plasmid. (A) The indicated strains were statically grown overnight in LB, subcultured in DMEM, and grown at 37°C to an OD_600_ of ∼0.2, and then IPTG was added (0.1 mM) and the bacteria were allowed to grow for an additional 1 h. The bacterial cultures were harvested, and normalized bacterial extracts were analyzed by Western blotting using an anti-Tir antibody. Nonspecific bands were used as a loading control. (B) HeLa cells were infected for 2.5 h with the above-mentioned strains, supplemented with IPTG (0.1 mM) to induce Tir expression. The infected cells were fixed, perforated, and stained with phalloidin rhodamine (which stains actin; red), and images were recorded. Bar, 25 μm. The white rectangle represents the enlarged area. (C) HeLa cells were infected as described in the legend to panel B with the indicted EPEC mutant supplemented with a Tir-expressing plasmid (gray bars) or not (black bars). The percentage of cells (*n* > 100) that contained pedestals (*n* > 20) was determined. Error bars represent the standard deviation from two biological repeats. Download FIG S5, TIF file, 2.7 MB. Copyright © 2019 Elbaz et al. 2019 Elbaz et al. This content is distributed under the terms of the Creative Commons Attribution 4.0 International license.

10.1128/mBio.02074-19.6 FIG S6 *LEE5* instability affects EPEC virulence factors. Wild-type EPEC, the *tir*ATG::AAA mutant, or a mutant from which *LEE* was deleted (the Δ*LEE* mutant) were statically grown overnight in LB, subcultured in DMEM, and grown at 37°C to an OD_600_ of ∼0.6, and then the bacterial cultures were harvested and normalized bacterial extracts and supernatant were analyzed by Western blotting using anti- EscJ, anti-EspF, anti-Map, anti-EspB, and anti-EspA antibodies. Nonspecific bands were used as a loading control (Ctrl). Download FIG S6, TIF file, 0.7 MB. Copyright © 2019 Elbaz et al. 2019 Elbaz et al. This content is distributed under the terms of the Creative Commons Attribution 4.0 International license.

### An increased level of CesT in the absence of Tir attenuates EPEC virulence.

**FIG 8:**
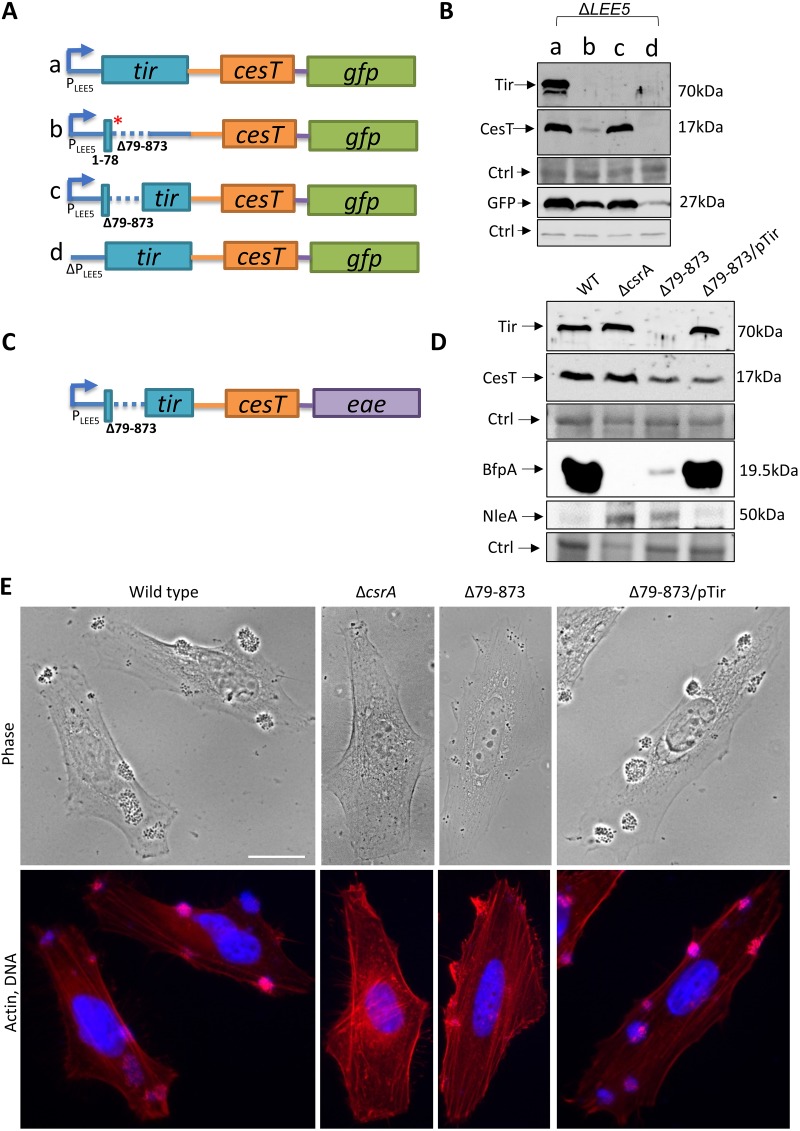
In-frame deletion of *tir* results in a pleiotropic phenotype. (A) Scheme showing plasmid-borne *LEE5* derivatives, including the wild type (row a), a mutant containing a deletion and a stop codon (row b), a mutant containing an in-frame deletion of *tir* (row c), and a mutant from which the *LEE5* promoter was deleted (row d) as a negative control. (B) The EPEC Δ*LEE5* mutant transformed with the plasmids in rows a to d in panel A was statically grown overnight in LB and subcultured in DMEM at 37°C to an OD_600_ ∼0.6. Proteins were then extracted and analyzed by Western blotting using anti-CesT/anti-Tir and anti-GFP antibodies. Nonspecific bands were used as a loading control (Ctrl). (C) Scheme showing the chromosomal *LEE5* operon containing an internal in-frame deletion within *tir*. (D) Wild-type EPEC or strains containing the Δ*csrA* mutant, the *tir*Δ79–873 mutant, or the *tir*Δ79–873 mutant complemented with a Tir-expressing plasmid, as shown in Fig. 5A, row a (NE7447, pTir), were statically grown overnight in LB. The bacteria were then subcultured in DMEM at 37°C to an OD_600_ of ∼0.5 and harvested. Normalized bacterial extracts were analyzed by Western blotting using antibodies against CesT/Tir, BfpA, or NleA. Nonspecific bands were used as a loading control (Ctrl). (E) The indicated strains were preactivated in DMEM for 3 h at 37°C and further utilized to infect HeLa cells for 30 min. The infected cells were fixed and stained with phalloidin rhodamine (which stains actin; red) and DAPI (which stains DNA; blue). Bar, 20 μm.

We next aimed at generating a mutant lacking Tir but expressing normal levels of CesT to evaluate how perturbation of the Tir/CesT ratio influences EPEC virulence. We first constructed a plasmid-borne *LEE5* operon containing a large in-frame internal deletion of *tir* (*tir*Δ79–873), expecting that this *LEE5* derivative would exhibit normal activity of the *LEE5* promoter, normal mRNA stability, and, consequently, normal CesT levels. Western blot analysis confirmed that, indeed, this was the case (Fig. 8A and B). We next constructed a similar deletion in the chromosomal *tir* of EPEC (Fig. 8C) and confirmed that the bacteria produced a significant amount of CesT, although the amount was not as large as that in wild-type EPEC (Fig. 8D). We expected that in the EPEC *tir*Δ79–873 mutant the absence of Tir would cause an increased level of free CesT. We further predicted that this increase in free CesT should influence the expression of virulence genes via inhibition of CsrA. We tested this prediction by assessing the amount of protein that was negatively (NleA) or positively (BfpA) regulated by CsrA (6). In agreement with this prediction, we noted that the EPEC *tir*Δ79–873 mutant showed an increase in NleA production and reduced BfpA production, phenocopying the *csrA* mutation (Fig. 8D). Importantly, in *trans* complementation with Tir restored the production of these proteins to wild-type levels (Fig. 8D). Furthermore, like the *csrA* mutant, EPEC *tir*Δ79–873 exhibited a poor formation of microcolonies and infectivity, a deficiency that was restored upon complementation with Tir (Fig. 8E). Taken together, these results show that Tir expression is essential for sequestering the coexpressed CesT, thus preventing the untimely repression of CsrA by CesT, which is deleterious for EPEC infectivity.

## DISCUSSION

The T3SS and associated effectors are critical for the virulence of A/E pathogens, and of these, the three most important effectors for host colonization are Tir, EspZ, and NleA (30–32). Interestingly, translocation of the first two and expression of the last one are CesT dependent (6, 8, 9, 33). CesT binds and stabilizes Tir and EspZ and targets them for translocation by the T3SS. Following Tir translocation, the liberated CesT antagonizes CsrA (Fig. 1A), an RNA-binding protein that functions as a global posttranscriptional regulator. The CesT-CsrA interaction results in remodeling of the expression pattern of numerous metabolic and virulence genes, including genes encoding the BFP, T3SS translocon proteins, and the NleA effector protein (6). These data emphasize the notion that CesT is a major regulator of EPEC virulence and is involved not only in effector stability and translocation but also in the posttranscriptional control of T3SS genes and metabolic genes of the host-attached EPEC. Furthermore, our results suggest that, in addition to its central role as an effector protein, Tir also functions as an antiregulator (i.e., anti-CesT) (Fig. 1A). These regulatory circuits are believed to be involved in the adaptation of the pathogen to a cell-adherent lifestyle and, possibly, to chronic infection.

To control the above-described regulatory circuits, the pathogen must manage the relative levels of effector-bound and free CesT, Tir, and CsrA in a tight and dynamic manner. In this study, we show that *tir* translation is essential for the stability of *cesT* mRNA, a mechanism that minimizes deviations from the optimal Tir/CesT ratio in planktonic bacteria. By placing stop codons along the *tir* coding region, we revealed a direct correlation between the length of the translated *tir* portions and the levels of *LEE5* mRNA stability. Accordingly, exclusion of the ATG start codon caused the maximal reduction in *cesT* mRNA levels. These data suggest that *tir* mRNA is intrinsically unstable throughout its length and that translation stabilizes it by preventing degradation. Our results concur with the notion that high ribosome densities mask RNase-sensitive sites residing within mRNA sequences and/or prevent RNase E from targeting mRNA 5′ ends (34, 35). However, our findings suggest that inactivation of RNase E is not sufficient to prevent the degradation of the untranslated *LEE5* mRNA, implying the involvement of an alternative RNase activity.

An additional mechanism that supports an optimal Tir/CesT ratio is the dependency of Tir stability on CesT, which guarantees that any Tir surplus is rapidly eliminated by degradation (9). Furthermore, being on the same transcriptional unit ensures coexpression of Tir and CesT from the same promoter. Interestingly, we found that efficient activation of this promoter is dependent on DNA sequences located downstream of it, within the 5′ UTR and coding region of *tir*.

We assessed the influence of *tir* inactivation on the expression of CsrA-regulated genes and EPEC infectivity using a tissue culture model. The mutated strains fell into two categories, of which the first type consisted of strains expressing no Tir but low levels of CesT. These mutants showed reduced effector translocation due to a lack of sufficient CesT levels. Furthermore, we could only partially complement these mutants in *trans*, since the complementing Tir failed to restore CesT production and thus underwent rapid degradation in the absence of a sufficient amount of CesT. The second mutation type contained an in-frame deletion of *tir* and showed nearly normal levels of CesT. In the absence of Tir, the produced CesT remained mostly free, repressing CsrA, and thus caused abnormal gene expression, including the premature production of NleA and the reduced expression of BFP. Thus, both strategies of *tir* inactivation had a pleiotropic effect. They not only caused a lack of Tir but also elicited secondary phenotypes due to either reduced levels of CesT or prematurely increased levels of free CesT. Our data suggest that avoiding such secondary effects, while generating a *tir* mutation, is challenging and requires the prudent construction of point mutations in the chromosomal *tir*.

Our data show that the dependence of *LEE5* mRNA stability on translation is required for maintaining a proper Tir/CesT ratio and thus enhances infection efficiency. This mechanism prevents an uncontrolled increase in CesT upon perturbations in Tir translation that may be mediated, for example, by the activity of sRNA. Interestingly, we recently identified an sRNA that downregulates *tir* translation and whose expression is upregulated under conditions that enhance *LEE* gene expression. Regulation of mRNA stability was also reported for other *LEE* operons, but how these contribute to the better fitness of the bacteria is yet to be elucidated. Among these operons is *LEE3* mRNA, which encodes structural proteins of the T3SS basal body (i.e., *escV*, *escN*) (36). Likewise, McAteer et al. (37) showed that YbeY, an endoribonuclease which is essential for the maturation of the 16S rRNA 3′ end, is required for the stability of multiple *LEE* transcripts.

In conclusion, in this report we have revealed the existence of integrated regulatory circuits that function to control the Tir/CesT ratio and to prevent premature overshooting of free CesT levels, which hamper CsrA regulation in the infecting pathogen.

## MATERIALS AND METHODS

### Strains, plasmids, primers, and basic procedures.

The bacterial strains, plasmids, primers, and antibodies used in this study are listed in Tables S1 and S2 in the supplemental material. The strains were constructed using the bacteriophage lambda Red system or by conjugation, as described previously (38). The plasmids were constructed using standard methods or isothermal assembly (39). Bacteria were grown in Luria-Bertani (LB) broth supplemented, when needed, with ampicillin (Amp; 100 μg/ml), streptomycin (Strp; 50 μg/ml), chloramphenicol (Cm; 25 μg/ml), tetracycline (Tet; 20 μg/ml), or kanamycin (Kn; 40 μg/ml). When indicated, different concentrations of IPTG (isopropyl-β-d-thiogalactopyranoside; Sigma) were added. To mimic infection conditions, bacteria were statically grown overnight in LB at 37°C and then subcultured by diluting 1:50 with Dulbecco’s modified Eagle medium (DMEM; Biological Industries) and grown for 5 h to an OD_600_ of ∼0.6. For complementation of the tir*Δ79–873 mutant, 0.5 mM IPTG was added 3 h after subculturing in DMEM to induce Tir or CesT expression. To ensure *LEE5* transcription, MG1655 strains were supplemented, when needed, with a plasmid expressing Ler (pGY2746) or LacI (pREP4; plasmid 223 in Table S1) and induced by 0.05 mM or 0.25 mM IPTG, respectively.

10.1128/mBio.02074-19.7 TABLE S1 Strains and plasmids used in this study. Download Table S1, DOCX file, 0.04 MB. Copyright © 2019 Elbaz et al. 2019 Elbaz et al. This content is distributed under the terms of the Creative Commons Attribution 4.0 International license.

### Western blot analysis.

Normalized bacterial extracts, boiled for 10 min in Laemmli buffer (Bio-Rad), were cleared and subjected to SDS-PAGE (typically using Bio-Rad 12% TGX precast gels). The loading amounts were normalized using the protein concentration or the OD of the used culture and further verified by recording the gels using stain-free imaging (Bio-Rad), Ponceau membrane staining (Sigma), or a nonspecific band as a loading control.

### Determination of GFP fluorescence intensity.

For E. coli K-12 MG1655, the strains were grown overnight in LB at 37°C under shaking conditions and then subcultured into M9 (Sigma) medium supplemented with 0.1% Casamino Acids (Difco) and 0.2% glucose. For EPEC cultures, the bacteria were grown in DMEM as indicated above; the cultures were then washed and suspended in phosphate-buffered saline (PBS). The fluorescence intensity of the GFP was measured (filter set at 485-nm excitation and 510-nm emission) and normalized according to the optical density (OD_600_) using a Spark 10M microplate reader (Tecan).

### Microscopy and infection.

HeLa cells were seeded in a 24-well plates (Nunc) at a density of ∼7 × 10^4^ cells per well and grown overnight in DMEM supplemented with 10% fetal calf serum (FCS; Biological Industries) and antibiotics (penicillin-streptomycin solution; Biological Industries). Next, the HeLa cells were infected with bacteria that had been statically grown overnight at 37°C (multiplicity of infection, ∼1:100), followed by 3 h of infection at 37°C in 5% CO_2_. Infecting strains were supplemented, if needed, with 0.5 mM IPTG. When indicated, the cells were infected with EPEC bacteria that had been preactivated for expression of the T3SS. For preactivation, the bacteria were diluted 1:100 in DMEM and grown statically at 37°C for 3 h. To terminate the infection, the cells were then fixed (3.7% formaldehyde in PBS), washed, perforated (PBS, 0.25% Triton X-100 for 10 min), washed, stained with phalloidin rhodamine (Sigma), and analyzed by fluorescence microscopy.

### Extraction of translocated Tir.

Translocated Tir was extracted as described previously (40), with some modifications. Briefly, HEK293T cells (∼5 × 10^6^ cells/100-mm plate) were infected with bacteria statically grown overnight at 37°C (multiplicity of infection, ∼1:100); IPTG (0.5 mM) was added as required. Infection was carried out for 3 h; the cells were then washed and scraped into 1 ml cold PBS and centrifuged gently (1,300 × *g*, 3 min), and the pellets were resuspended in 200 μl cold lysis buffer (1% Triton X-100 and complete inhibitor in PBS). Bacteria, host cell nuclei, and the cytoskeleton were pelleted by centrifugation (20,000 × *g* for 3 min). The resulting supernatant contained the cytoplasm and membrane proteins of the host cells.

### CesT stability assay.

EPEC Δ*LEE5* or Δ*cesT* mutants were transformed with a plasmid expressing CesT via an IPTG-regulated promoter (pSK6194). The bacterial cultures were statically grown overnight in LB at 37°C, subcultured by 1:50 dilution with DMEM, and incubated statically at 37°C. Upon reaching an OD_600_ of ∼0.2, 0.5 mM IPTG was added to stimulate CesT expression. After an additional 30 min, chloramphenicol (100 μg/ml) was added to stop translation. Samples were then collected at different time points, proteins were extracted, and normalized amounts were analyzed by Western blotting using anti-CesT antibody.

### RNA extraction and reverse transcription-PCR.

Total RNA was extracted with the TRIzol reagent (Sigma) according to the manufacturer’s instructions. The quality of RNA was assessed by determination of the *A*_260_/*A*_280_ ratio. RNA (1.5 μg) was treated with RQ1 DNase I (Promega) at a concentration of 1 U/μg RNA for 30 min at 37°C. DNase I was inactivated by adding 1 μl of stop solution and heating the samples for 15 min at 65°C. DNA digestion was verified by PCR, using primers 1952 and 1953. For *cesT* mRNA, cDNA was synthesized with a Verso cDNA synthesis kit (Thermo) and amplified using primers 3863 and 3864 (Table S2). For *tir* mRNA, cDNA was synthesized with a qPCRBIO high-quality cDNA synthesis kit (catalog number PB30.11-10; PCR Biosystems) as described by the manufacturer and amplified using primers 3876 and 3877 (Table S2). cDNA was quantified by real-time PCR using a SYBR green mix (Absolute SYBR Green ROX mix; Thermo) with a Rotor Gene 6000 real-time PCR machine (Corbett) according to the manufacturer’s instructions. Specific primer pairs were designed according to the *Guidelines for Amplicon and Primer Design* (http://www.tamar.co.il/tamar-laboratory-supllies/guidelines-amplicon-primer-design/). The level of 16S rRNA (*rrsB*) was used to normalize the expression data for *cesT*. The relative amount of cDNA was calculated by the standard curve method, which was obtained by PCR of serially diluted genomic DNA as the templates and analyzed using Rotor Gene analysis software (version 6.0).

### Examination of RNase E involvement in *LEE5* degradation.

For examination of RNase E involvement in *LEE5* degradation, we used a previously described method (41), with slight modifications. Briefly, E. coli K-12 (MG1655) or an isogenic strain containing the *rne3071* mutation (an RNase E temperature-sensitive mutant [RNase E^ts^]) was transformed with the plasmids shown in Fig. 5C, rows a and c, and further supplemented with a LacI-expressing plasmid (pREP4), enabling tight control over the expression of recombinant *tir*. The bacteria were then grown overnight in LB (30°C) and subcultured in M9 medium (Sigma) supplemented with 0.1% Casamino Acids (Difco) and 0.2% glucose. The bacteria were grown at 30°C to an OD_600_ of 0.3, and then the temperature was shifted to 42°C for 30 min. Next, IPTG (0.25 mM) was added and OD_600_-normalized GFP expression levels were monitored by fluorimetry in real time at 5-min intervals (a filter set at 485-nm excitation and 510-nm emission) using a Spark 10M microplate reader (Tecan).
