# Supplementary figures and images for: Control of Type III Secretion System Effector/Chaperone Ratio Fosters Pathogen Adaptation to Host-Adherent Lifestyle
Source: mBio. 2019 Sep 17;10(5):e02074-19. doi: 10.1128/mBio.02074-19 (PMC6751064; doi:10.1128/mBio.02074-19)

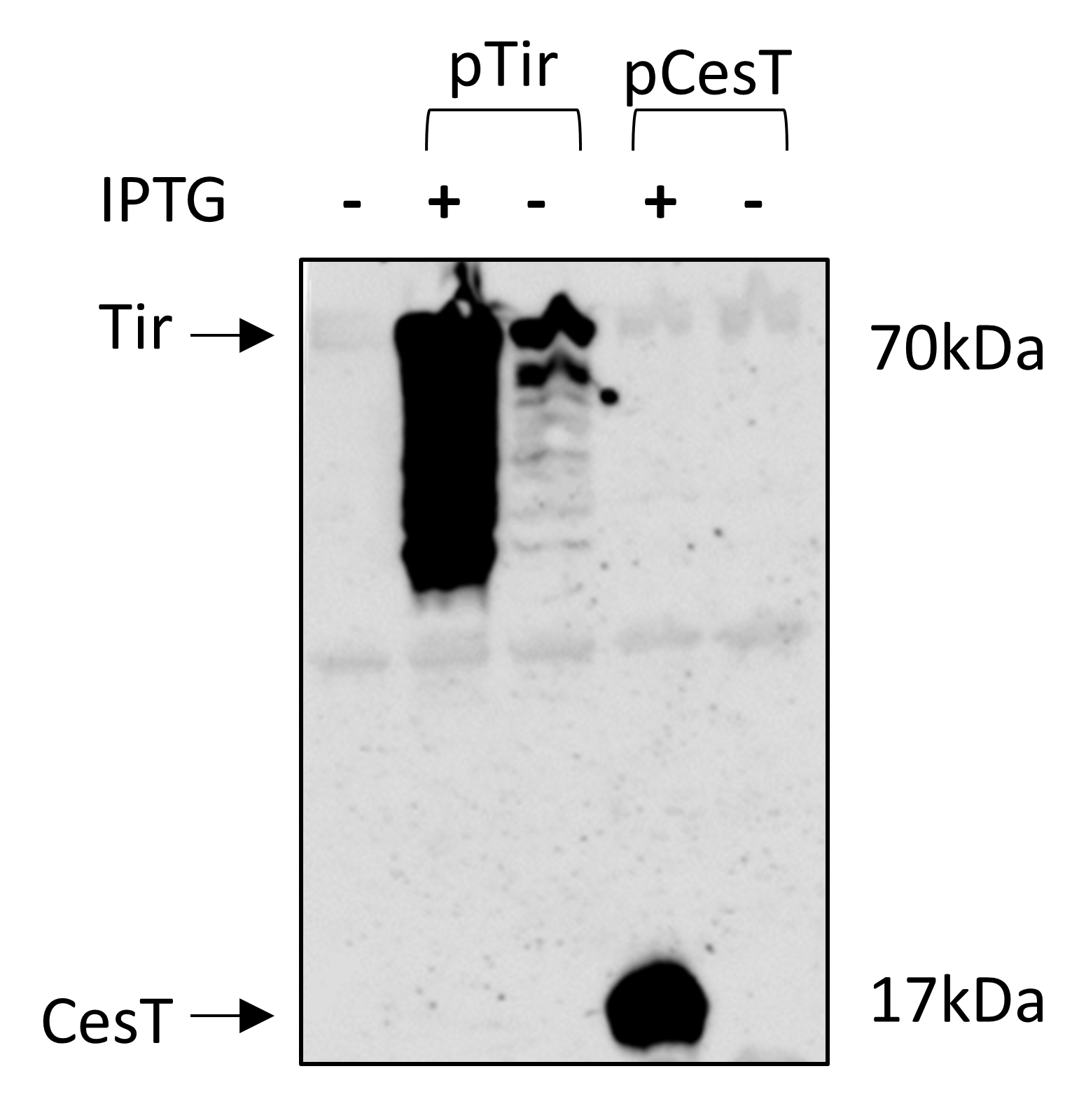

Supplement: FIG S1 [file mBio.02074-19-sf001.tif]

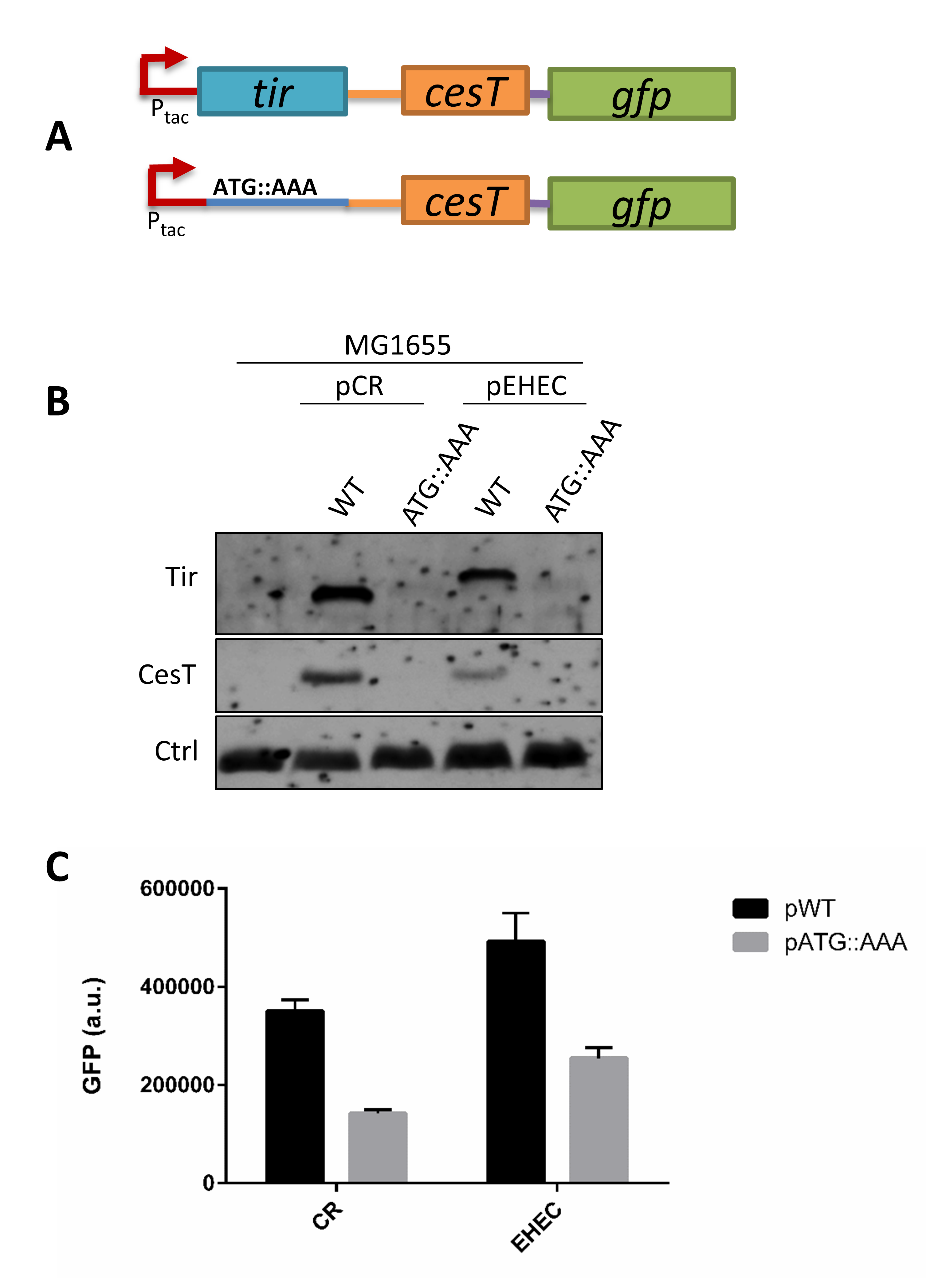

Supplement: FIG S2 [file mBio.02074-19-sf002.tif]

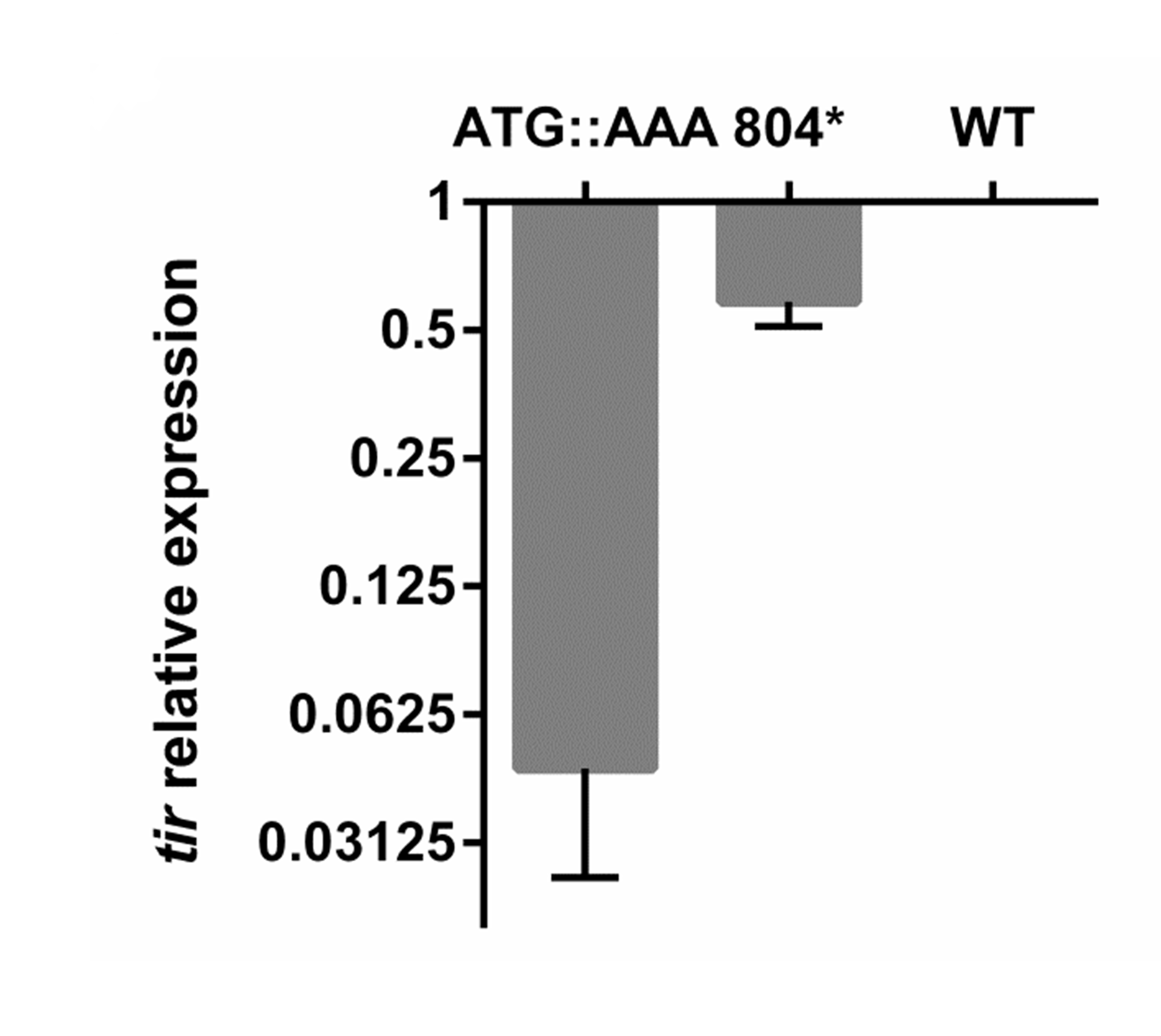

Supplement: FIG S3 [file mBio.02074-19-sf003.tif]

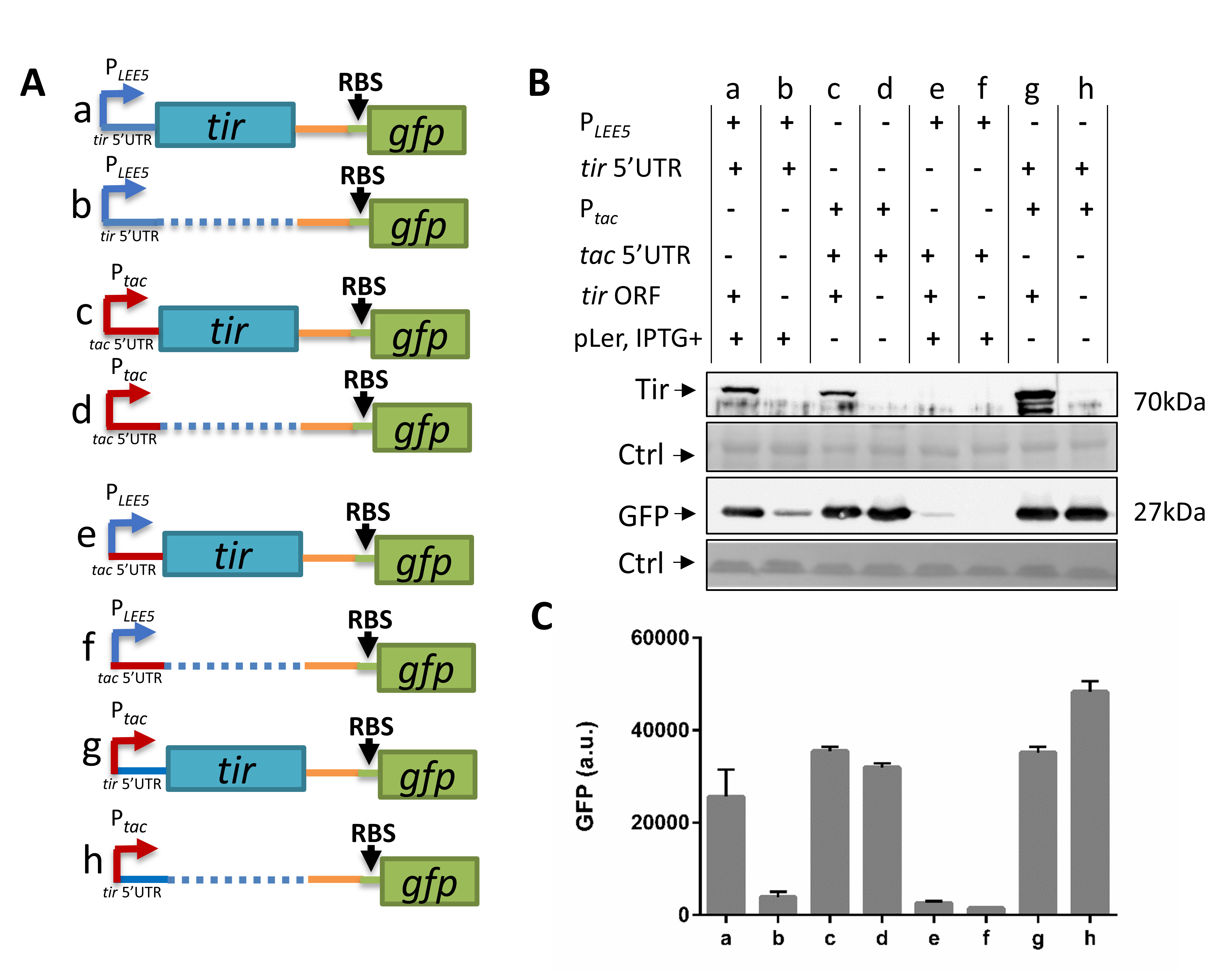

Supplement: FIG S4 [file mBio.02074-19-sf004.tif]

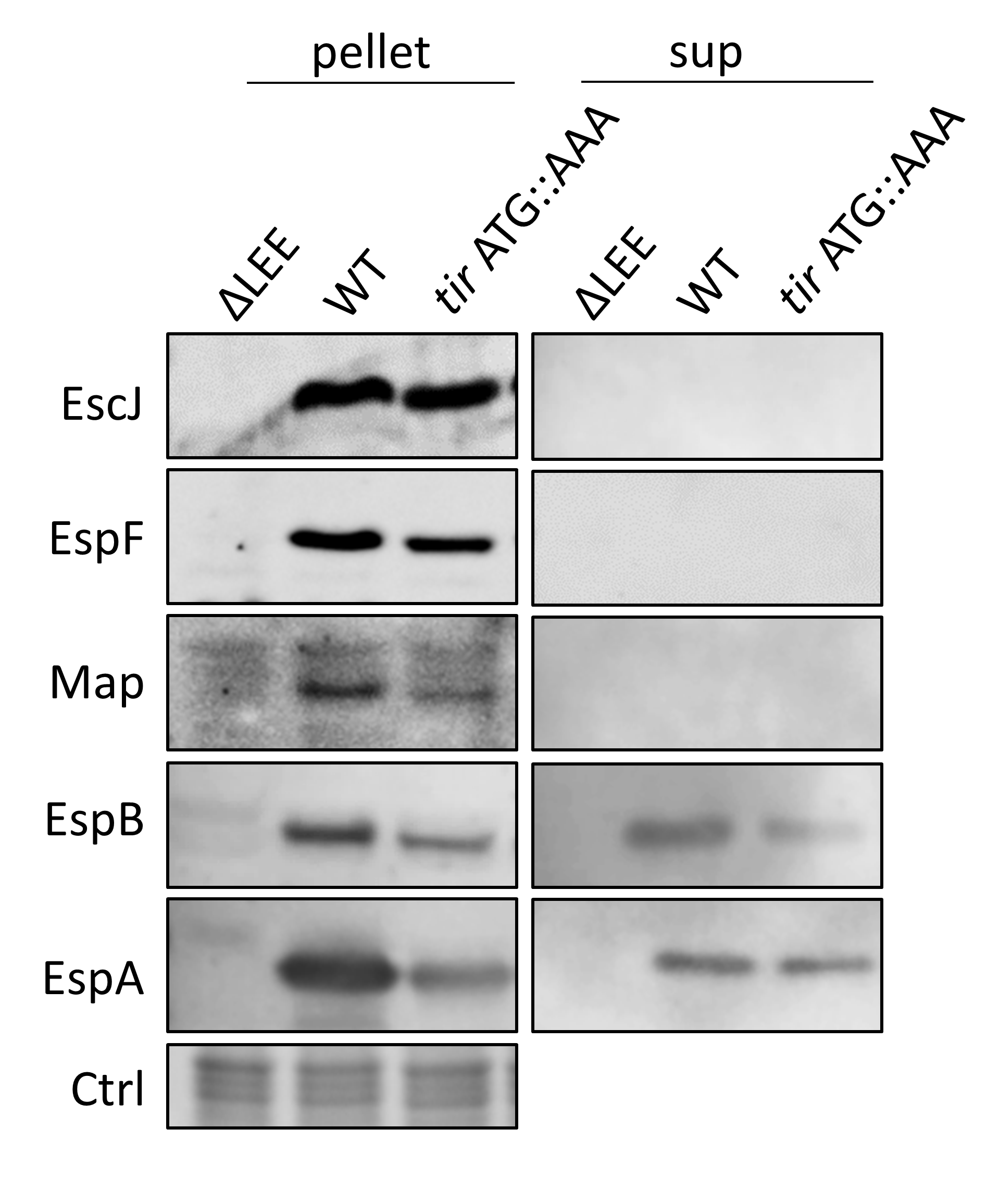

Supplement: FIG S6 [file mBio.02074-19-sf006.tif]
