# Supplementary material for: Control of Type III Secretion System Effector/Chaperone Ratio Fosters Pathogen Adaptation to Host-Adherent Lifestyle
Source: mBio. 2019 Sep 17;10(5):e02074-19. doi: 10.1128/mBio.02074-19 (PMC6751064; doi:10.1128/mBio.02074-19)
Supplement: TABLE S2 [file mBio.02074-19-st002.docx]

**Table S2: Primers and antibodies used in this study**

Table S2A: Primers used to create deletions and insertions

| **Number** | **Sequence** | **Usage** |
| --- | --- | --- |
| 3849 | TGTGATTTTT TAGTTGGAAA TACAG  ACATG CATTTCTGGT GCGTTATTTT GCATTCCGGGGA TCCGTCGACC | Creation of NE7632 |
| 3850 | CCG ACT AAA AGA TAC ATT TTA GCC GGA CAA TGA TTA GAT ATT TAT TAA ATG TGT AGG CTG GAG CTG CTT C | Creation of NE7632 |
| 3618 | CTC TAA CTG AGA AGG CGA TTG | Verification of NE7632, Creation of NE7630,NE7631,NE7845, NE7566, |
| 3570 | GCGAAACGATCCTCATCCTGT | Verification of NE7632 |
| 66 | CGG TGC CCT GAA TGA ACT GC | Verification of NE7632 |
| 1456 | CGC CAG GGG TTG CAG GAT GGG | Verification of NE7632 |
| 3632 | TGT TTG TGA AGG TAG TGG CGG | Creation of NE7565, NE7566 |
| 3832 | CCGCCACTACCTTCACAAACATAAGACGGCGCGGCACGGGGAG | Creation of NE7565 |
| 3740 | TATAATTTCTCTTAATTGATTTATAATAACAGAAGTTCAAGAGTTAC | Creation of NE7565, NE7630 NE7564, NE7630 ,NE7631,NE7845, NE 7566 |
| 3320 | GGT ATG GCC CAC CAT TCT CTG CA | Verification of NE7565,NE7630,NE7631, NE7845, YS8527 |
| 3831 | ATATAAAGGA ACGTGTCAAA TTTCTAAATA AAAGGATATA TGTAAA CCT ATT GGT AAC CTT GGT AAT AAT GTA AAT GG | Creation of NE7564 |
| 631 | GTTTAGTCCAACGGCAATGGTAGG | Verification of NE7564,NE7630,NE7631, NE7845 |
| 1666 | CGTATCAACGTCCTTGGGTTG | Verification of NE7564, NE7630,NE7631, NE7845 |
| 3852 | TGT AAC GCT AAC CTC CAA ACC A | Creation of NE7630 |
| 3851 | TGGTTTGGAG GTTAGCGTTACATAATTA AGT CCT CAA GAA TGG AGC AGC | Creation of NE7630 |
| 3854 | GGT TGT AGG ATC ATC CGG TTC CGG TG | Creation of NE7631 |
| 3853 | CA CCGGAACCGG ATGATCCTACAACCTAAACCGAT CCTGATCAGG CCGC | Creation of NE7631 |
| 3744 | CAAATTTCTAAATAAAAGGATATATGTATGCCTAGTCCTCAAGAATGGAGCAGCTTGC | Verification of NE7564, NE7630,NE7631, NE7845 |
| 3928 | TGTTGTTGTAGTAGTTGTCTGTTCTG | Creation of NE7845 |
| 3927 | CAGAACAGACAACTACTACAACAACATAACATACGGTAGTGCAGCAACAGACCGG | Creation of NE7845 |
| 3825 | AAAATAGAGG AAATTAGCTC AAGCGATAAT AAACATTATT ACGCCGGAAG A ATGCGTA AAGGAGAAGA ACTTTTCACT G | Creation of NE7557, NE7559, NE7570, NE7572 |
| 1849 | TTATTTGTAGAGCTCATCCATGC | Creation of NE7557,NE7558 NE7559, NE7560 |
| 3827 | CATGGCA TGGATGAGCT CTACAAATAAATT CCG GGG ATC CGT CGA CC | Creation of NE7557,NE7558 NE7559, NE7560 |
| 3828 | CACAATGAGTTAGAATGAGTAGTAAATTAATACTAATAAATAAGTTGTGTAGGCTGGAGCTGCTTC | Creation of NE7557,NE7558 NE7559, NE7560 |
| 1498 | CAACGTTGCA GCATGGGTAA CTCTTG | Verification of NE7557, NE7558, NE7559, NE7560, NE7576 |
| 1159 | GTGCCCATTAACATCACCATC | Verification of NE7557, NE7558, NE7559, NE7560, NE7570, NE7576 |
| 3826 | GAAATTAGCTC AAGCGATAAT AAACATTATT ACGCCGGAAGATAA TGTCTAGATTT AAGAAGGAGA TATACATATGCG | Creation of NE7558, NE7560, NE7570, NE7576, NE7578 |
| 3619 | GTTTGGGCTCCACCACAATG | Creation of NE7570, NE7576, NE7572, NE7578 |
| 3788 | CTAA ATAAAAGGAT ATATGT ATATATCTGT GAGTATTTAG TTGAGGTTGG GG | Creation of NE7566 |
| 4210 | GTCTCACGCGAAATCTTTGAACAGAATTTTAGCACCGCGAAAATCAGTGATATCAAGGCTCTAACTGAGAAGGCGATTG | Creation of YS8527 |

Table S2B: Primers used to create the plasmids

| **Number** | **Sequence** | **Usage** |
| --- | --- | --- |
| 3616 | CATTGTGGTGGAGCCCAAACATGCGTAAAGGAGAAGAACTTTTCACTGG | pNE7199, pNE7200 |
| 3617 | AATCGCCTTCTCAGTTAGAGGGCGCGCCTACCTGTGAC | pNE7199 pNE7200,  pNE7865,  pNE7866 |
| 3618 | CTC TAA CTG AGA AGG CGA TTG | pNE7199 pNE7200,  pNE7865,  pNE7866 |
| 3619 | GTTTGGGCTCCACCACAATG | pNE7199 pNE7200 |
| 3631 | CCGCCAC TACCTTCACA AACAAAGAACCCTGA GAACCAGAAA GTTAACATC | pNE7241 |
| 3632 | TGT TTG TGA AGG TAG TGG CGG | pNE7241 |
| 3635 | CTC TAA CTG AGA AGG CGA TTG CAG ATGCCTATTGGTAACCT TGGTAATAAT G | pNE7263 |
| 3636 | CTG CAA TCG CCT TCT CAG TTA GAG | pNE7263 |
| 3740 | TATAATTTCTCTTAATTGATTTATAATAACAGAAGTTCAAGAGTTAC | pNE7447, pNE7437 |
| 3741 | TATAAATCAATTAAGAGAAATTATAGTCTAGA  TTTAAGAAGGAGATATACATATGCGTAAAGG  AGAAGAACTTTTCACTG | pNE7447, pNE7437 |
| 3831 | ATATAAAGGA ACGTGTCAAA TTTCTAAATA AAAGGATATA TGTAAA CCT ATT GGT AAC CTT GGT AAT AAT GTA AAT GG | pNE7638 |
| 3789 | ACA TAT ATC CTT TTA TTT AGA AAT TTG ACA CGT TCC | pNE7638 |
| 3868 | CAA TTTCACACAG GAAACAGAAT TCATG CCT ATT GGT AAC CTT GGT AAT AAT GTA AAT G | pNE7665, pNE7666 |
| 3596 | GGCGCGCCTACCTGTGAC | pNE7665, pNE7666,  pNE7667,  pNE7869,  pNE7870 |
| 3866 | CCGT CACAGGTAGG CGCGCCGATCCAGCTTATCGACTGCAC | pNE7665, pNE7666,  pNE7667, |
| 1636 | GAATTCTGTTTCCTGTGTGAAATTG | pNE7665, pNE7666, pNE7667,  pNE8249, pNE8250,  pNE8251,  pNE8252 |
| 3869 | CAA TTTCACACAG GAAACAGAAT TCAAA CCT ATT GGT AAC CTT GGT AAT AAT GTA AAT G | pNE7667 |
| 3788 | CTAA ATAAAAGGAT ATATGT ATATATCTGT GAGTATTTAG TTGAGGTTGG GG | pNE7626 |
| 3789 | ACA TAT ATC CTT TTA TTT AGA AAT TTG ACA CGT TCC | pNE7626 |
| 3870 | CAA TTTCACACAG GAAACAGAAT TC ATA TAT CTG TGA GTA TTT AGT TGA GGT TG | pNE7668 |
| 3972 | CA AAAATTATAC TGTGATTTATTTGGTTTA AAT TGT GAG CGG ATA ACA ATT TCA CAC AG | pNE7865, pNE7866 |
| 3718 | TAA ACC AAA TAA ATC ACA GTA TAA TTT TTG ATG CAA GC | pNE7865,  pNE7866 |
| 4273 | CAA TTTCACACAGGAAACAGAAT TC ATGCCTATTG GTAATCTTGG TAATAATAAT ATAAG | pNE8249 |
| 4274 | CAA TTTCACACAGGAAACAGAAT TCAAACCTATTG GTAATCTTGG TAATAATAAT ATAAGTAAC | pNE8250 |
| 4275 | GTT ATA GGC TCC ACC ACA ATG AG | pNE8249, pNE8250 |
| 4276 | CTCATTGT GGTGGAGCCT ATAACATG CGT AAA GGA GAA GAA CTT TTC ACT G | pNE8249, pNE8250 |
| 4278 | CAA TTTCACACAGGAAACAGAAT TCATGCCTATTG GTAATCTTGG TCATAATC | pNE8251 |
| 4279 | CAA TTTCACACAGGAAACAGAAT TCAAACCTATTG GTAATCTTGG TCATAATCCC | pNE8252 |
| 4280 | GTT ATG GCT CCA CCA CAA TGA GTT AG | pNE8251, pNE8252 |
| 4281 | CT AACTCATTGT GGTGGAGCCA TAACATG CGT AAA GGA GAA GAA CTT TTC ACT G | pNE8251, pNE8252 |
| 3789 | ACA TAT ATC CTT TTA TTT AGA AAT TTG ACA CGT TCC | pNE7626 |
| 3972 | CA AAAATTATAC TGTGATTTATTTGGTTTA AAT TGT GAG CGG ATA ACA ATT TCA CAC AG | pNE7865, pNE7866 |
| 3718 | TAA ACC AAA TAA ATC ACA GTA TAA TTT TTG ATG CAA GC | pNE7865,  pNE7866 |
| 3973 | CAATTAATC ATCGGCTCGT ATAATGTGTG GTGC TCA GTT GTT TTA TCG GCT GCA TAC | pNE7869, pNE7870 |
| 3866 | CCGT CACAGGTAGG CGCGCCGATCCAGCTTATCGACTGCAC | pNE7869, pNE7870 |
| 3974 | CCA CAC ATT ATA CGA GCC GAT G | pNE7869, pNE7870 |

Table S2C: Primers used for qPCR

| **Number** | **Sequence** | **Usage** |
| --- | --- | --- |
| 1952 | CAG AGA TGA GAA TGT GCC TTC GGG | Amplification of *rrsB* gene (1) |
| 1953 | CCG CTG GCA ACA AAG GAT AAG  G | Amplification of *rrsB* gene (1) |
| 3863 | TGATTTATGGCGTCTGTGGA | Amplification of *cesT* gene.  This study |
| 3864 | TTTCAGGGGTAGCATCATCG | Amplification of *cesT* gene.  This study |
| 3876 | GCAGAAGACGCTTCTCTGAATA | Amplification of *tir* gene (2) |
| 3877 | CCCAACTTCAGCATATGGATTA | Amplification of *tir* gene (2) |

Table S2D: Antibodies used in this study and working conditions

| **Antibody** | **Source** | **Dilution** |
| --- | --- | --- |
| Anti-CesT | James Kaper | 1:1000 |
| Anti Tir | Gad Frankel | 1:2000 |
| Anti-GFP | Clontech 632460 | 1:1000 |
| Peroxidase AffiniPure Goat Anti-Rabbit IgG (H+L) | Jackson 111-035-003 | 1:10000 |
| Alkaline phosphatase, Goat Anti-Rabbit IgG | Sigma A-3687 | 1:10000 |
| Anti-Tubulin alpha | Sigma T5168 | 1:1000 |
| Anti-mouse IgG, HRP-linked Antibody | Cell Signaling #7076 | 1:10000 |
| Anti-Intimin | Gad Frankel | 1:2500 |
| Anti-EscJ | Lab collection | 1:1000 |
| Anti-EspF | Lab collection | 1:2500 |
| Anti-Map | Lab collection | 1:1000 |
| Anti-EspB | Gad Frankel | 1:2500 |
| Anti-EspA | Gad Frankel | 1:5000 |
| Anti-BfpA | Michael Donnenberg | 1:1000 |

**References**

1. Park JH, Lee KH, Kim TY, Lee SY. 2007. Metabolic engineering of Escherichia coli for the production of L-valine based on transcriptome analysis and in silico gene knockout simulation. Proc Natl Acad Sci U S A 104:7797-802.

2. Leverton LQ, Kaper JB. 2005. Temporal expression of enteropathogenic Escherichia coli virulence genes in an in vitro model of infection. Infect Immun 73:1034-43.
