# Supplementary material for: Control of Type III Secretion System Effector/Chaperone Ratio Fosters Pathogen Adaptation to Host-Adherent Lifestyle
Source: mBio. 2019 Sep 17;10(5):e02074-19. doi: 10.1128/mBio.02074-19 (PMC6751064; doi:10.1128/mBio.02074-19)
Supplement: TABLE S1 [file mBio.02074-19-st001.docx]

**Table S1: Strains and plasmids used in this study**

Table S1A: Strains

| **Name (Number in our collection)** | **Description** | **Reference or source** |
| --- | --- | --- |
| E2348/69 (1) | EPEC wild type isolate O127:H6 | J. Kaper |
| EPEC*nleA*-_RBS_*gfp* (EM4624) | E2348/69 containing integrated pEM4618 (p*nleA-_RBS_gfp*) | (1) |
| EPEC*nleA*-*gfp* (EM4620) | E2348/69 containing integrated pEM4617 (p*nleA-gfp*) | (1) |
| EM2018 | E2348/69 Δ*cesT::kn* | (2) |
| CX2168 | E2348/69 ∆*eae::kn* | (3) |
| SM10λpir (5176) | *E. coli, thi-1, th, leu, tonA, lacY, supE, recA::RP4-2-Tc::Mu, λpir* | Lab collection |
| NN5343 | E2348/69 Δ*tir* containing integrated pEM4617 (p*nleA-gfp*) | This study |
| NN5350 | E2348/69 Δ*tir* containing integrated pEM4618 (p*nleA-_RBS_gfp*) | This study |
| NN5385 | NN5343 containing pEM2181 (p*tir)* | This study |
| NN6249 | EM4620 containing pNN6237 (p*cesT)* | This study |
| GY4811 | Dh5a | Lab collection |
| NN6237 | Dh5a containing pNN6237 (p*cesT)* | (1) |
| EM 2181 | Dh5a containing pEM2181 (p*tir)* | (1) |
| EM 3458 | E2348/69 Δ*tir* | (3) |
| EM 3470 | EM 3458 containing pEM2181 (p*tir)* | This study |
| SK6233 | EM2018 containing pSK6194 (p*cesT*) | This study |
| YL5768 | *XTL634* tet-sacB cassette containing strain | (4) |
| NE7449 | E2348/69 *tir::tet-sacB* | This study |
| NE7632 | E2348/69 Δ*LEE5*::kn | This study |
| NE7679 | NE7632 containing pSK6194 (p*cesT*) | This study |
| NE7565 | E2348/69 tir containing stop codon at position 78 | This study |
| NE7943 | NE7632 containing pNE7199 (*tir,cesT,gfp)* | This study |
| NE7944 | NE7632 containing pNE7200 (*tir*Δ*79-873*,cesT,gfp)* | This study |
| NE7945 | NE7632 containing pNE7241 (*tirΔ*79-873*,cesT,gfp)* | This study |
| NE7946 | NE7632 containing pNE7263 (Δp*LEE5*_,_*tir,cesT,gfp)* | This study |
| NE7564 | E2348/69 *tir* ATG::AAA | This study |
| NE7630 | E2348/69 *tir* containing stop codon at position 402 | This study |
| NE7631 | E2348/69 *tir* containing stop codon at position 804 | This study |
| NE7845 | E2348/69 *tir* containing stop codon at position 1200 | This study |
| NE7557 | E2348/69 containing *cesT-gfp* translational fusion | This study |
| NE7558 | E2348/69 containing *cesT-_rbs_gfp* transcriptional fusion | This study |
| NE7559 | EM3458 containing *cesT-gfp* translational fusion | This study |
| NE7560 | EM3458 containing *cesT-_rbs_gfp* transcriptional fusion | This study |
| NE7570 | NE7564 containing *cesT-gfp* translational fusion | This study |
| NE7576 | NE7564 containing *cesT-_rbs_gfp* transcriptional fusion | This study |
| NE7624 | MG1655 | Lab collection |
| NE7625 | MG1655 containing pGY2746 (p*ler)* | This study |
| NE7655 | NE7625 containing pNE7447 (P*_LEE5_*, *tir* 5'UTR,*tir*,*tir*-*cesT* intergenic region (IR), *_rbs_gfp)* | This study |
| NE7656 | NE7625 containing pNE7437 (P*_LEE5_*, *tir* 5'UTR*, tir*Δ*79-873, *tir*-*cesT* IR, *_rbs_gfp)* | This study |
| NE7657 | NE7625 containing pNE7638 (P*_LEE5_*, *tir* 5'UTR*, tir* ATG::AAA, *tir*-*cesT* IR, *_rbs_gfp)* | This study |
| NE7665 | NE7624 containing pNE7665 (P_tac_, tac 5'UTR,*tir*,*tir*-*cesT* IR, *_rbs_gfp)* | This study |
| NE7666 | NE7624 containing pNE7666 (P_tac_, tac 5'UTR*, tir*Δ*79-873, *tir*-*cesT* IR, *_rbs_gfp* | This study |
| NE7667 | NE7624 containing pNE7667 (P_tac_, tac 5'UTR, *tir* ATG::AAA, *tir*-*cesT* IR, *_rbs_gfp)* | This study |
| NE7668 | NE7624 containing PNE7668 (P_tac_, tac 5'UTR, *Δtir*,*tir*-*cesT* IR, *_rbs_gfp)* | This study |
| NE8249 | NE7624 containing pNE8249 (P_tac_, tac 5'UTR, *Citrobacter rodentium* (CR) *tir, tir-cesT IR, CesT, cesT-eae IR, gfp)* | This study |
| NE8250 | NE7624 containing pNE8250 (P_tac_, tac 5'UTR, CR *tir* ATG::AAA*, tir-cesT IR, CesT, cesT-eae IR, gfp)* | This study |
| NE8251 | NE7624 containing pNE8249 (P_tac_, tac 5'UTR, *enterohemorrhagic E.coli* (EHEC) *tir, tir-cesT IR, CesT, cesT-eae IR, gfp)* | This study |
| NE8252 | NE7624 containing pNE8250 (P_tac_, tac 5'UTR, EHEC *tir* ATG::AAA*, tir-cesT IR, CesT, cesT-eae IR, gfp)* | This study |
| GY4368 | *Citrobacter rodentium* ICC-168 | G. Frankel |
| NE8253 | GY4368 containing pNE8249 | This study |
| NE8254 | GY4368 containing pNE8250 | This study |
| #1280 | EHEC EDL933 slt1-,slt2- | J. Leung |
| NE8255 | #1280 containing pNE8251 | This study |
| NE8256 | #1280 containing pNE8252 | This study |
| NE7659 | MG1655 *rne*3071,zce-726::Tn10 | (5) |
| #223 | XL-1Blue containing pREP4 | Qiagen |
| NE8164 | NE7659 containing pREP4 | This study |
| NE8165 | NE8164 containing pNE7665 | This study |
| NE8166 | NE8164 containing pNE7667 | This study |
| NE7566 | E2348/69 containing complete Δ*tir* ORF | This study |
| NE7572 | NE7566 containing *cesT-gfp* translation fusion | This study |
| NE7578 | NE7566 containing *cesT-_rbs_gfp* transcription fusion | This study |
| NE7626 | MC1061 containing pNE7626 | This study |
| NE7658 | NE7625 containing pNE7626 (P*_LEE5_*, *tir* 5'UTR,*Δtir*,*tir*-*cesT* IR, *_rbs_gfp)* | This study |
| NE7867 | NE7625 containing pNE7865 (P*_LEE5_*, tac 5'UTR,*tir*, *tir*-*cesT* IR, *_rbs_gfp)* | This study |
| NE7868 | NE7625 containing pNE7866 (P*_LEE5_*, tac 5'UTR, *Δtir*,*tir*-*cesT* IR, *_rbs_gfp)* | This study |
| NE7869 | NE7624 containing pNE7869 (P_tac,_ *tir* 5'UTR, *tir, tir*-*cesT* IR, *_rbs_gfp)* | This study |
| NE7870 | NE7624 containing pNE7670 P_tac,_ *tir* 5'UTR, *Δtir, tir*-*cesT* IR, *_rbs_gfp)* | This study |
| NE8158 | NE7564 containing pEM2181 | This study |
| NE8159 | NE7565 containing pEM2181 | This study |
| NE8160 | NE7630 containing pEM2181 | This study |
| NE8161 | NE7631 containing pEM2181 | This study |
| NE8162 | NE7845 containing pEM2181 | This study |
| NN5898 | E2348/69 Δ*csrA::cm* | (1) |
| YS8527 | E2348/69 *tir* Δ79-873 in-frame deletion | This study |
| YS8537 | YS8527 containing pNE7447 | This study |

Table S1B: Plasmids

| **Name (Number in our collection)** | **Description** | **Reference or source** |
| --- | --- | --- |
| pKD46 (p811) | Contains the *λ red* genes. | (6) |
| pKD13  **(**p814) | Template for the kanamycin resistance cassette. | (6) |
| pREP4  (p223) | Constitutively LacI^q^ expressing plasmid | Qiagen |
| pCesT (pNS6237) | A pSA10 derivative containing *cesT* between EcoRI and SalI sites, cm resistance | (1) |
| p*nleA-RBSgfp* (pEM4618) | A pGP704 derivative containing EPEC *nleA-gfp* transcriptional fusion *(nleA-RBSgfp*). | (1) |
| p*nleA-gfp* (pEM4617) | A pGP704 derivative containing EPEC *nleA-gfp+* translational fusion (*nleA-gfp*). | (1) |
| p*nleA-RBSgfp* (pNN5782) | pZS*1 derivative containing *nleA-RBSgfp+* transcriptional fusion including the *nleA* regulatory region.  used as template for *gfp*/*_rbs_gfp* | (1) |
| pTir-His (pME2181) | A pSA10 derivative expressing Tir, C-terminally tagged with *6xHIs* | (1) |
| pCesT  (pSK6194) | A pSA10 derivative containing *cesT* between EcoRI and SalI sites, amp resistance | (1) |
| pZS*12rGFP (p5656) | A pZ derivative containing pSC101* ORI and ampicillin resistance cassette. | (7) |
| pNE6655 | A pZ derivative containing pSC101* ORI cm resistance cassette and *_rbs_gfp*, used as template for pSC101* vector | This study |
| pGY2746 | A pSA10 derivative expressing Ler | (8) |
| pNE7943 | A pZS*1 derivative containing P*_LEE5_*,*tir*,*tir-cesT* IR ,cesT,*cesT-eae* IR,gfp | This study |
| pNE7944 | A pZS*1 derivative containing P *_LEE5_*, *tir**Δ79-873, tir-cesT IR, cesT,cesT-eae IR,gfp | This study |
| pNE7945 | A pZS*1 derivative containing P*_LEE5_*, *tir*Δ79-873, *tir-cesT* IR, cesT,*cesT-eae* IR, gfp | This study |
| pNE7946 | A pZS*1 derivative containing ΔP*_LEE5_*, *tir*,*tir-cesT* IR, cesT, *cesT-eae* IR, gfp | This study |
| pNE7447 | A pZS*1 derivative containing P*_LEE5_*, *tir* 5'UTR, *tir*, *tir*-*cesT* intergenic region (IR), *_rbs_gfp* | This study |
| pNE7437 | A pZS*1 derivative containing P*_LEE5_*, *tir* 5'UTR*, tir*Δ*79-873, *tir*-*cesT* IR, *_rbs_gfp* | This study |
| pNE7638 | A pZS*1 derivative containing P*_LEE5_*, *tir* 5'UTR*, tir* ATG::AAA, *tir*-*cesT* IR, *_rbs_gfp* | This study |
| pNE7665 | A pZS*1 derivative containing P_tac_, tac 5'UTR, *tir*,*tir*-*cesT* IR, *_rbs_gfp* | This study |
| pNE7666 | A pZS*1 derivative containing P_tac_, tac 5'UTR*, tir*Δ*79-873,*tir*-*cesT* IR, *_rbs_gfp* | This study |
| pNE7667 | A pZS*1 derivative containing P_tac_, tac 5'UTR, *tir* ATG::AAA, *tir*-*cesT* IR, *_rbs_gfp* | This study |
| pNE7668 | A pZS*1 derivative containing P*_LEE5_*, *tir* 5'UTR, *Δtir*, *tir*-*cesT* IR, *_rbs_gfp* | This study |
| pNE8249 | A pZS*1 derivative containing P_tac_, tac 5'UTR, *Citrobacter rodentium* (CR) *tir, tir-cesT IR, CesT, cesT-eae IR, gfp* | This study |
| pNE8250 | A pZS*1 derivative containing P_tac_, tac 5'UTR, CR *tir* ATG::AAA*, tir-cesT IR, CesT, cesT-eae IR, gfp* | This study |
| pNE8251 | A pZS*1 derivative containing P_tac_, tac 5'UTR, *enterohemorrhagic E.coli* (EHEC) *tir, tir-cesT IR, CesT, cesT-eae IR, gfp* | This study |
| pNE8252 | A pZS*1 derivative containing P_tac_, tac 5'UTR, *enterohemorrhagic E.coli* (EHEC) *tir* ATG::AAA*, tir-cesT IR, CesT, cesT-eae IR, gfp* | This study |
| pNE7626 | A pZS*1 derivative containing P*_LEE5_*, *tir* 5'UTR, *Δtir*, *tir*-*cesT* IR, *_rbs_gfp* | This study |
| pNE7865 | A pZS*1 derivative containing P*_LEE5_*, tac 5'UTR, *tir*, *tir*-*cesT* IR, *_rbs_gfp* | This study |
| pNE7866 | A pZS*1 derivative containing P*_LEE5_*, tac 5'UTR, *Δtir*, *tir*-*cesT* IR, *_rbs_gfp* | This study |
| pNE7869 | A pZS*1 derivative containing P_tac,_ *tir* 5'UTR, *tir, tir*-*cesT* IR, *_rbs_gfp* | This study |
| pNE7870 | A pZS*1 derivative containing P_tac,_ *tir* 5'UTR, *Δtir, tir*-*cesT* IR, *_rbs_gfp* | This study |

**References**

1. Katsowich N, Elbaz N, Pal RR, Mills E, Kobi S, Kahan T, Rosenshine I. 2017. Host cell attachment elicits posttranscriptional regulation in infecting enteropathogenic bacteria. Science 355:735-739.

2. Li M, Rosenshine I, Yu HB, Nadler C, Mills E, Hew CL, Leung KY. 2006. Identification and characterization of NleI, a new non-LEE-encoded effector of enteropathogenic Escherichia coli (EPEC). Microbes Infect 8:2890-8.

3. Mills E, Baruch K, Aviv G, Nitzan M, Rosenshine I. 2013. Dynamics of the type III secretion system activity of enteropathogenic Escherichia coli.

4. Li XT, Thomason LC, Sawitzke JA, Costantino N, Court DL. 2013. Positive and negative selection using the tetA-sacB cassette: recombineering and P1 transduction in Escherichia coli. Nucleic Acids Res 41:e204.

5. Carpousis AJ, Van Houwe G, Ehretsmann C, Krisch HM. 1994. Copurification of E. coli RNAase E and PNPase: evidence for a specific association between two enzymes important in RNA processing and degradation. Cell 76:889-900.

6. Datsenko KA, Wanner BL. 2000. One-step inactivation of chromosomal genes in Escherichia coli K-12 using PCR products. Proc Natl Acad Sci U S A 97:6640-5.

7. Lutz R, Bujard H. 1997. Independent and tight regulation of transcriptional units in Escherichia coli via the LacR/O, the TetR/O and AraC/I1-I2 regulatory elements. Nucleic Acids Res 25:1203-10.

8. Yerushalmi G, Nadler C, Berdichevski T, Rosenshine I. 2008. Mutational analysis of the locus of enterocyte effacement-encoded regulator (Ler) of enteropathogenic Escherichia coli. J Bacteriol 190:7808-18.
